# Unraveling Inhibition Mechanism of Structurally Distinct Flavonoids Against α-Amylase and α-Glucosidase: Insights from Multi-Spectroscopic and In Silico Analyses

**DOI:** 10.3390/foods15203731

**Published:** 2026-10-20

**Authors:** Meng Li, Zhishen Mu, Kefan Li, Zhanmei Jiang

**Affiliations:** 1 Department of Food Science and Engineering, College of Pharmacy, Heilongjiang University of Chinese Medicine, Harbin 150030, China; wzjk1995lm@126.com; 2 Key Laboratory of Dairy Science, Ministry of Education, College of Food Science, Northeast Agricultural University, Harbin 150030, China; 3 Inner Mongolia Enterprise Key Laboratory of Dairy Nutrition, Health & Safety, Inner Mongolia Mengniu Dairy (Group) Co., Ltd., Huhhot 011500, China

**Keywords:** flavonoids, α-amylase, α-glucosidase, inhibition mechanisms

## Abstract

Type II diabetes mellitus is a growing global health burden, and postprandial hyperglycemia is an independent risk factor for diabetic complications. α-amylase (αA) and α-glucosidase (αG) are key digestive enzymes involved in carbohydrate hydrolysis and represent important targets for investigating the regulation of postprandial carbohydrate digestion. In this study, baicalein (BA), liquiritigenin (LI), and puerarin (PU) were systematically investigated against αA and αG. Results showed that the three flavonoids inhibited αA and αG in a dose-dependent manner, with the inhibitory potency following the order of PU > BA > LI. Kinetic analyses revealed that three flavonoids exhibited mixed-type inhibition toward αA but non-competitive inhibition toward αG. Fluorescence results indicated that flavonoids quenched intrinsic fluorescence of αA and αG through a static mechanism with approximately one binding site, and the binding was spontaneous and driven predominantly by hydrogen bonding and van der Waals forces. Multi-spectroscopic analyses further demonstrated that flavonoid binding induced conformational rearrangements of enzymes, manifested by the burial of aromatic residues into more hydrophobic microenvironments and the conversion of secondary structures from random coil to ordered α-helix and β-sheet. Moreover, in silico analysis revealed that hydrogen bonding and van der Waals forces were the key driving forces between αA/αG and BA/LI/PU. Furthermore, abundant hydroxyl groups of PU provided additional interaction sites for hydrogen-bond formation, contributing to its superior inhibitory efficiency. These findings provide molecular-level evidence for the structure-dependent inhibition of αA/αG by flavonoids under the present in vitro conditions and provide a basis for further investigation of their physiological relevance.

## 1. Introduction

Diabetes mellitus (DM) is a globally prevalent chronic metabolic disorder, with type II diabetes mellitus (T2DM) accounting for more than 90% of all diabetic cases. The major clinical manifestation of T2DM is abnormal postprandial hyperglycemia [1]. Persistent elevation of postprandial blood glucose levels can contribute to the development of various complications, including stroke, cardiovascular diseases, lower limb dysfunction, and visual impairment. Dietary carbohydrates play a crucial role in regulating postprandial glucose fluctuations [2]. However, insulin resistance or insufficient insulin secretion in individuals with T2DM often leads to excessive carbohydrate digestion and subsequent rapid glucose release, thereby exacerbating postprandial hyperglycemia [3]. During carbohydrate digestion, α-amylase (αA) and α-glucosidase (αG) serve as two key digestive enzymes responsible for the sequential hydrolysis of complex polysaccharides into absorbable glucose, thereby directly influencing postprandial glycemic responses [4,5]. During carbohydrate digestion, α-amylase (αA) and α-glucosidase (αG) are responsible for the sequential hydrolysis of complex carbohydrates into absorbable sugars and therefore play important roles in regulating the rate of carbohydrate digestion. Consequently, inhibition of αA and αG has been widely investigated as an in vitro approach for modulating carbohydrate hydrolysis. Several clinically used enzyme inhibitors, including acarbose, voglibose, and miglitol, inhibit carbohydrate-hydrolyzing enzymes, although their administration may be accompanied by gastrointestinal adverse effects [6]. These observations have stimulated interest in identifying naturally occurring compounds capable of inhibiting αA and αG.

Among naturally occurring bioactive compounds, polyphenols have received considerable interest due to their diverse biological activities and potential inhibitory effects against carbohydrate-hydrolyzing enzymes. Numerous studies have demonstrated that polyphenols interact with αA and αG primarily through non-covalent interactions, including hydrogen bonding and hydrophobic interactions, leading to alterations in enzyme conformation, masking of catalytic regions, or interference with substrate binding [7,8,9]. The inhibitory efficiency of polyphenols is highly dependent on their chemical structures and functional group distributions. For instance, hydrophobic interactions between the aromatic rings of phenolic acids (hydroxybenzoic acid and ferulic acid) and tryptophan residues within αA have been reported to facilitate enzyme inhibition [10]. Among polyphenolic compounds, flavonoids represent one of the most extensively investigated subclasses due to their widespread distribution in plants and remarkable structural diversity. In addition to their well-established antioxidant, anti-inflammatory, lipid-regulating, and cardioprotective properties, flavonoids have attracted considerable interest because of their diverse biological activities, including reported effects on carbohydrate metabolism in different experimental models [11].

Structurally, flavonoids possess a characteristic C6-C3-C6 carbon skeleton and can be classified into several subclasses, including flavones, flavonols, flavanones, isoflavones, and chalcones, according to differences in C-ring oxidation patterns, B-ring attachment positions, and conjugated systems [12]. Previous studies have demonstrated that structural characteristics of flavonoids are closely associated with their inhibitory activities against αA and αG. For instance, flavonoids with similar structures (apigenin, scutellarein, hispidulin, and nepetin) inhibited enzyme hydrolysis by reducing the catalytic activity of αA [13]. Furthermore, flavonoids extracted from dandelion [14], Nelumbo nucifera leaves [15], and taxifolin [16] could form non-covalent complexes with enzymes, thereby inhibiting the activities of αA and αG. Specifically, variations in hydroxyl group number and position, glycosylation degree, and conjugation patterns may significantly influence their binding affinity, interaction modes, and inhibition mechanisms. However, the molecular mechanisms underlying how structural diversity among flavonoids determines their binding behaviors, inhibition modes, and regulatory effects on digestive enzymes remain insufficiently understood.

In this study, three naturally occurring flavonoids with distinct structural characteristics, namely baicalein (BA), liquiritigenin (LI), and puerarin (PU), representing flavone, flavanone, and isoflavone subclasses, respectively, were selected as model compounds. A multidimensional approach was employed to systematically investigate the non-covalent interaction mechanisms between BA/LI/PU and αA/αG. Specifically, enzymatic inhibition assays, inhibition kinetic analysis, fluorescence spectroscopy, UV-Vis absorption spectroscopy, circular dichroism spectroscopy, Fourier-transform infrared spectroscopy, molecular docking, and molecular dynamics simulations were integrated to elucidate the molecular mechanisms by which structurally diverse flavonoids regulate digestive enzyme activity. This study aimed to clarify the relationships between flavonoid structural characteristics, enzyme-binding behavior, and inhibition mechanisms toward αA and αG under defined in vitro conditions.

## 2. Materials and Methods

### 2.1. Chemicals and Reagents

Human pancreatic α-amylase (αA, 10 U/mg) and yeast-derived α-glucosidase (αG, 40 U/mg) were purchased from Shanghai Yuanye Bio-Technology Co., Ltd. (Shanghai, China). Baicalein (BA), liquiritigenin (LI), and puerarin (PU), obtained as aqueous-extracted flavonoid preparations, were purchased from Xi’an Ruilin Bio-Technology Co., Ltd. (Xi’an, China). All other chemical reagents and solvents utilized were of analytical grade.

### 2.2. Preparation of Enzyme-Flavonoid Complexes

Flavonoid solutions (BA/LI/PU) were prepared in 0.1 mol/L phosphate buffer (pH 6.8) containing dimethyl sulfoxide (DMSO, 10% *v*/*v*) and vortexed thoroughly before use. Flavonoid solutions (0.4 mL) at concentrations of 0, 2, 4, 8, 16, and 32 μg/mL were mixed with 2 mL αA/αG, respectively, and incubated at 37 °C for 20 min. The final DMSO concentration in the enzyme-flavonoid mixtures was approximately 1.67% (*v*/*v*). All flavonoid solutions were freshly prepared and visually inspected before use, and no visible precipitation or turbidity was observed under the experimental conditions. The mixtures were subsequently lyophilized for further analyses and designated as αA-BA, αA-LI, αA-PU, αG-BA, αG-LI and αG-PU, respectively.

### 2.3. Inhibition Assays of α-Amylase

According to the procedures described in the previous method [17], 0.5 mL of flavonoid solutions of three different structures at various concentrations (0, 0.3, 0.6, 0.9, 1.2, 1.5, 1.8, 2.1 and 3 mg/mL) were mixed with 0.5 mL of αA (dissolved in 0.1 mol/L PBS, pH 6.8). Subsequently, the mixture was incubated at 37 °C for 15 min. Soluble starch solution (0.1 mL, 5 mg/mL) was then introduced into the mixture under continuous stirring. The resulting reaction solution was further incubated at 37 °C for 5 min. Thereafter, 0.75 mL of 3,5-dinitrosalicylic acid was added to the mixture, which was then heated in a boiling water bath for 8 min to terminate the reaction. The mixture was allowed to cool to room temperature, followed by dilution with the addition of 3 mL of PBS. Finally, the absorbance of the hydrolysates was measured at 540 nm using a UV-Vis spectrophotometer (UV-6100, Shanghai, China). Subsequently, the αA inhibition rate was calculated through the following formula:

$$\mathrm{\alpha A}\;\mathrm{inhibitory}\;\mathrm{rate}\;\left(\%\right)=\left(1-\frac{A_{s}-A_{\mathrm{SB}}}{A_{C}-A_{\mathrm{CB}}}\right)\times 100\%$$

where, A_S_, A_SB_, A_C_, and A_CB_ represented the absorbance values of the sample reaction group (containing enzymes), the sample blank group (without enzymes), the control reaction group (containing PBS), and the control blank group (without samples and enzymes), respectively.

### 2.4. Inhibition Assays of α-Glucosidase

According to the previously established method [7], 1 mL of flavonoid solutions of three different structures at various concentrations (0, 0.3, 0.6, 0.9, 1.2, 1.5, 1.8, 2.1 and 3 mg/mL) were separately mixed with 1 mL of αG (dissolved in 0.1 mol/L PBS, pH 6.8). The mixture was incubated at 37 °C for 20 min. Subsequently, 1 mL of p-nitrophenyl-α-D-glucopyranoside was added to the mixture, and incubation was continued at 37 °C for 30 min. The reaction was then terminated by adding 2 mL of Na_2_CO_3_ solution (1 mol/L). The absorbance of the reaction mixture was determined at 405 nm using a UV-Vis spectrophotometer (UV-6100). Subsequently, the αG inhibition rate was also calculated using the following formula:

$$\mathrm{\alpha G}\;\mathrm{inhibitory}\;\mathrm{rate}\;\left(\%\right)=\left(1-\frac{{A_{1}}^{\prime }-A_{1}}{{A_{0}}^{\prime }-A_{0}}\right)\times 100\%$$

where, A_0_, A_0_′, A_1_, and A_1_′ denoted the absorbance values of the sample reaction group (containing enzymes), the sample blank group (without enzymes), the control reaction group (containing PBS), and the control blank group (without samples and enzymes), respectively.

### 2.5. Enzyme Inhibition Kinetics Analysis

Enzyme inhibition kinetics were determined following a method described by Zhu et al. [6]. For αA, soluble starch (2.5, 5.0, 7.5, and 10.0 mg/mL) was used as the substrate, and the released reducing sugars were quantified by the DNS method at 540 nm. For αG, p-nitrophenyl-α-D-glucopyranoside (pNPG, 0.5, 1.0, 2.0, and 4.0 mmol/L) was used as the substrate, and released p-nitrophenol (pNP) was measured at 405 nm. BA, LI, and PU concentrations were 0, 0.3, 0.6, and 0.9 mg/mL for the kinetic assays. The initial reaction rates (V_0_) obtained at different substrate and inhibitor concentrations were globally fitted by nonlinear regression using the following competitive, noncompetitive, and mixed inhibition models:

Competitive inhibition:

$$V_{0}=\frac{V_{\max}\left[S\right]}{K_{m}\left(1+\frac{\left[I\right]}{k_{\mathrm{ic}}}\right)+\left[S\right]}$$

Noncompetitive inhibition:

$$V_{0}=\frac{V_{\max}\left[S\right]}{\left(1+\frac{\left[I\right]}{k_{i}}\right)\times \left(K_{m}+\left[S\right]\right)}$$

Mixed inhibition:

$$V_{0}=\frac{V_{\max}\left[S\right]}{K_{m}\left(1+\frac{\left[I\right]}{k_{\mathrm{ic}}}\right)+\left[S\right]\left(1+\frac{\left[I\right]}{k_{\mathrm{iu}}}\right)}$$

where, V_0_ was the initial reaction rate, V_max_ was the maximum reaction rate, K_m_ was the Michaelis constant. [S] and [I] denoted the concentration of substrate (mg/mL) and inhibitor (mM). k_ic_ and k_iu_ described inhibitor binding to free enzyme and the enzyme-substrate complex, respectively. k_i_ was the inhibition constant in the noncompetitive model. The three models were compared using the corrected Akaike information criterion (AICc) and residual analysis.

Furthermore, the inhibition types and inhibition constants of the three structurally distinct flavonoids on the enzymes were determined using the Lineweaver-Burk (LB) equation and its transformations. The half-maximal inhibitory concentration (IC_50_) was obtained from the fitted dose-response curve of inhibition rate versus flavonoid concentration. Acarbose was analyzed under the same experimental conditions and used as a positive control.

### 2.6. Intrinsic Fluorescence Spectroscopy Analysis

The intrinsic fluorescence of the samples was determined at 298.15 K, 303.15 K and 310.15 K using a fluorescence spectrophotometer (F-7000, Tokyo, Japan) with the following parameters: excitation wavelength λ_ex_ = 280 nm, emission wavelength λ_em_ = 290–450 nm, excitation and emission slit widths of 2.5 and 5 nm, respectively, a scanning speed of 240 nm/s, and an operating voltage of 650 V [18]. Furthermore, the intrinsic fluorescence of the samples was scanned at 298.15, 303.15, and 310.15 K under identical parameters for subsequent fluorescence quenching analysis. The fluorescence spectra of BA, LI, and PU alone at the corresponding concentrations were recorded under the same experimental conditions and used as ligand blanks. The intrinsic fluorescence contribution of each flavonoid was subtracted from the corresponding enzyme-flavonoid spectrum before subsequent analysis. The ligand-background-corrected fluorescence intensities were further corrected for the inner-filter effect.

The quenching constants were calculated using the Stern-Volmer equation to determine the quenching mechanism:

*F*_0_/*F* = 1 + *K_q_τ*_0_[*Q*] = 1 + *K_sv_*[*Q*]

where, F_0_ and F denoted the fluorescence intensities of the biomacromolecule in the absence and presence of the quencher, respectively. K_q_ was the bimolecular quenching rate constant. τ_0_ was the average fluorescence lifetime prior to the addition of the quencher (typically taken as 10^−8^ s). K_sv_ represented the Stern-Volmer quenching constant, and [Q] was the concentration of the quencher.

The Stern-Volmer plot represented a single class of fluorophores in the protein, all of which were equally accessible to the quencher, implying that only a single quenching mechanism (either dynamic or static quenching) occurs. Under the static quenching mechanism, the relationship between the binding constants of the biomacromolecule and the quencher could be calculated using the double-logarithmic equation:

$$\log\left[\frac{F_{0}-F}{F}\right]=\log K_{a}+n\log\left[Q\right]$$

where, K_a_ and n denote the binding constant and the number of binding sites between the biomacromolecule and the quencher, respectively. [Q] was the concentration of the quencher.

### 2.7. Calculation of Thermodynamic Parameters

The interaction forces between the biomacromolecule and small molecules were evaluated based on the Van’t Hoff and thermodynamic equations. The Van’t Hoff equation was expressed as follows:

*lnK_a_* = −Δ*H*/*RT* + Δ*S*/*R*

The thermodynamic equation was given as follows:

Δ*G* = Δ*H* − *T*Δ*S*

where, △H and △S represented the enthalpy change and entropy change, respectively. △G denoted Gibbs free energy. R was the gas constant (8.314 J/mol K). T signified the absolute temperature.

### 2.8. Synchronous Fluorescence Spectroscopy Analysis

The synchronous fluorescence spectra of αA and αG interacting with the three flavonoids were scanned at 298 K within the range of 230–450 nm using a fluorescence spectrophotometer (F-7000, Tokyo, Japan), with the parameters set at λ_ex_ = 285 nm and △λ (△λ = λ_em_ − λ_ex_) fixed at 15 or 60 nm.

### 2.9. UV-Vis Absorption Spectroscopy Analysis

The complex solutions were transferred into a quartz cuvette at room temperature. UV-Vis absorption profiles of the samples were subsequently acquired using a UV-Vis spectrophotometer (EVOLUTION201, Madison, WI, USA) across a wavelength range of 240–600 nm [8].

### 2.10. Circular Dichroism Spectroscopy Analysis

Following the experimental protocols described in previous literature [4], circular dichroism (CD) spectra were analyzed. The complex solutions were introduced into a quartz cuvette with a 0.1 cm path length at room temperature. CD spectra of the samples were then scanned using a CD spectrometer (Chirascan, Surrey, UK) over a wavelength range of 190–260 nm.

### 2.11. Fourier Transform Infrared Spectroscopy Analysis

In accordance with previously established methodologies [8], Fourier transform infrared (FTIR) spectra were determined. The lyophilized complexes were placed directly onto an attenuated total reflection accessory. FTIR spectral data of samples were subsequently collected using an FTIR spectrometer (Nicolet iS50, Madison, WI, USA) within a wavenumber range of 4000–500 cm^−1^. Background correction was conducted against the ambient air prior to sample analysis.

### 2.12. Molecular Docking

Molecular docking was performed using AutoDock Vina 1.2.7. The crystal structures of human pancreatic α-amylase (αA, PDB ID: 5KEZ) and Saccharomyces cerevisiae isomaltase (used as an αG-related structural model, PDB ID: 3A4A) were obtained from the Protein Data Bank. The molecular structures of BA (CID: 5281605), LI (CID: 114829), and PU (CID: 5281807) were retrieved from PubChem. Protein and ligand structures were converted into PDBQT format for docking, with Gasteiger charges assigned and ligand rotatable bonds defined. The docking grid centers were set at (−8.424, 21.720, −19.518) Å for αA and (26.207, −5.161, 19.320) Å for αG, with corresponding grid dimensions of 47.453 × 64.553 × 48.308 Å and 47.790 × 63.666 × 57.267 Å, respectively. The exhaustiveness was set to 32, and the docking poses were ranked according to the Vina scoring function. The resulting protein-ligand interactions were visualized and analyzed using PyMOL (version 2.5). An additional redocking assessment was performed using the crystallographic α-D-glucose (GLC) ligand in the 3A4A structure. The native GLC ligand was redocked using Schrödinger Glide 2023-1 with standard precision (SP) and the OPLS_2005 force field. The docking box was centered at (16.963, −8.759, 19.586) Å, with dimensions of 26 × 26 × 26 Å. The resulting GLC pose was compared with its crystallographic conformation using the root-mean-square deviation (RMSD) of 12 corresponding heavy atoms. Both the RMSD calculated in the fixed protein coordinate frame and that obtained after independent ligand superposition were evaluated. For 5KEZ, conventional small-molecule redocking was not performed because its co-crystallized inhibitor is a peptide rather than a small molecule.

### 2.13. Molecular Dynamics Simulation

Protein-ligand complexes were subjected to molecular dynamics (MD) simulations using GROMACS 2025.5 with AMBER99SB-ILDN and General Amber Force Field (GAFF) for proteins and ligands, respectively. Each complex was solvated with TIP3P water in an orthorhombic periodic box with a minimum solute-to-box distance of 1.2 nm. Na^+^ and Cl^−^ ions were added to neutralize the systems and achieve a NaCl concentration of 0.15 mol/L. Following steepest-descent energy minimization, the systems were equilibrated for 100 ps under the NVT ensemble and 100 ps under the NPT ensemble at 300 K and 1 bar. Three independent 100 ns simulations were performed for each complex under periodic boundary conditions using a 2 fs time step, with coordinates saved every 0.01 ns. Temperature and pressure were controlled using the V-rescale thermostat and Parrinello-Rahman barostat, respectively. Short-range van der Waals and Coulomb interactions used a 1.0 nm cut-off, while long-range electrostatic interactions were treated using the particle-mesh Ewald method. Bond constraints were applied using LINCS and SETTLE. RMSD, RMSF, radius of gyration (Rg), solvent-accessible surface area (SASA), hydrogen bonds, secondary structure, and free-energy landscapes were analyzed, with equilibration assessed using PELT change-point detection. Binding free energies and per-residue energy contributions were calculated using gmx_MMPBSA 1.7.0 based on 15 conformations sampled from each trajectory.

### 2.14. Statistical Analysis

Triplicate measurements were performed for each sample, and the outcomes were averaged and reported as mean ± SD. Statistical comparisons among groups were made by one-way ANOVA using IBM SPSS Statistics 27 (IBM Corp., Armonk, NY, USA), with *p* < 0.05 considered significant. Charts and fitted curves were generated in Origin 2025 (OriginLab Corp., Northampton, MA, USA).

## 3. Results and Discussions

### 3.1. Inhibition Activity of Flavonoids Against αA and αG

**Figure 1:**
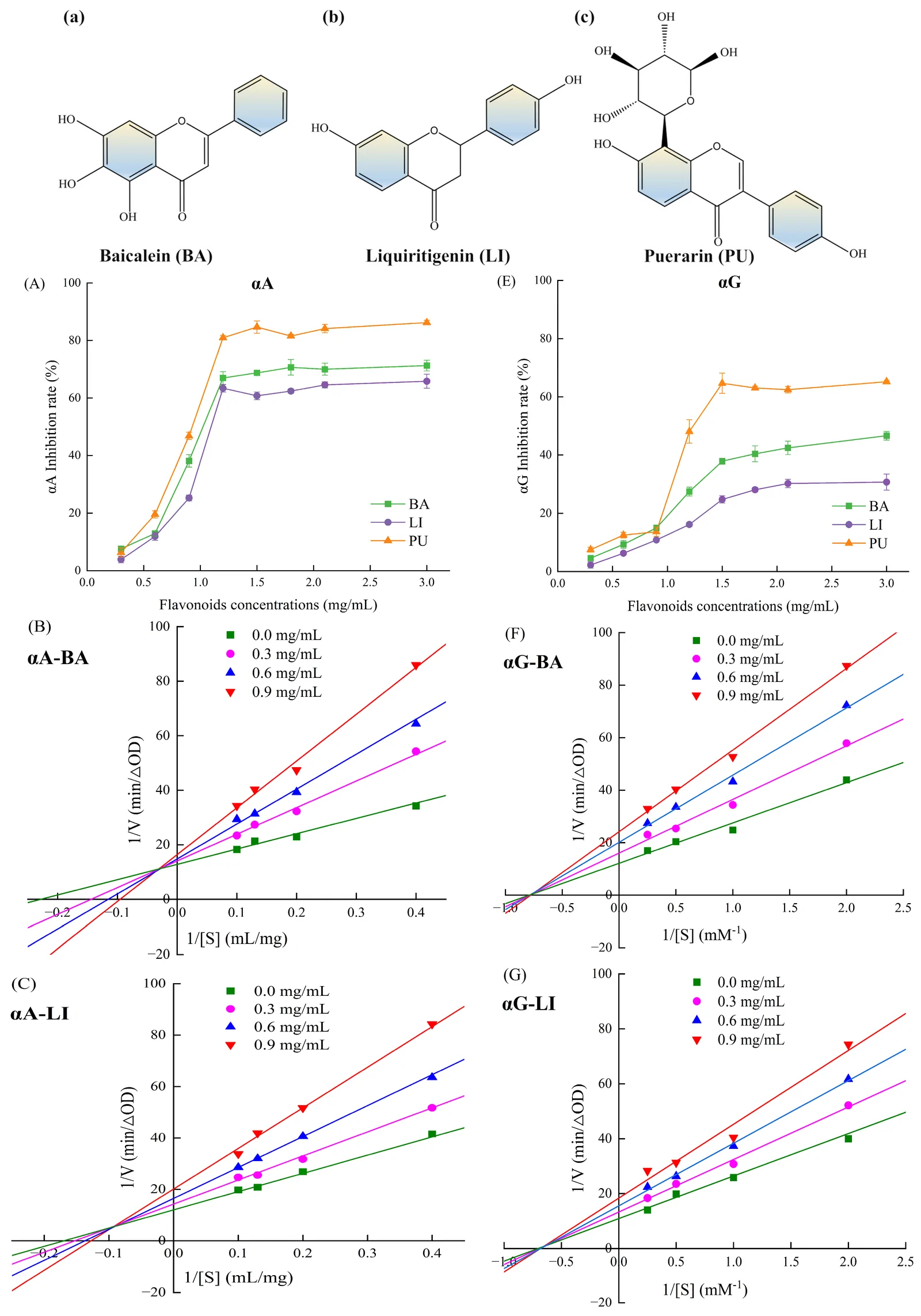

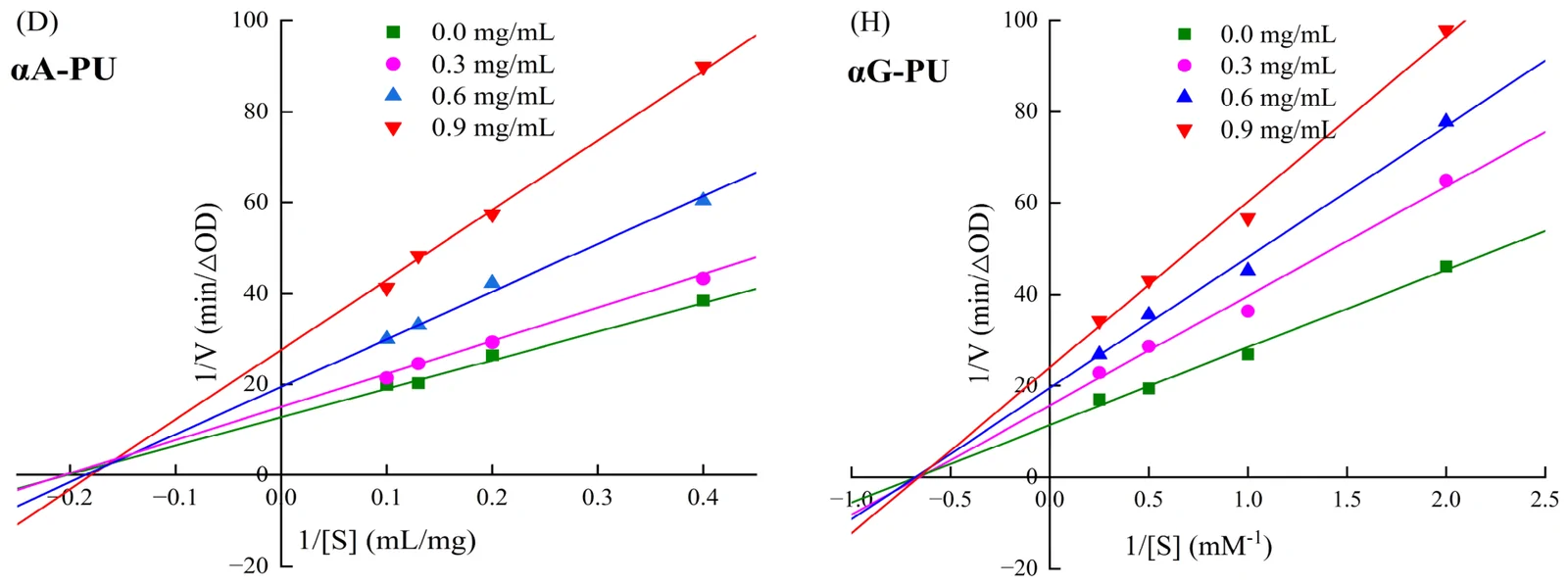
Structural diagrams of baicalein (**a**), liquiritigenin (**b**) and puerarin (**c**). Inhibition curves of BA, LI and PU on αA (**A**) and αG (**E**). Lineweaver-Burk plots of inhibition on αA (**B**–**D**) and αG (**F**–**H**) by BA, LI and PU.

During the process of human digestion, dietary starch is sequentially hydrolyzed by αA and αG, ultimately releasing glucose into the bloodstream. Flavonoids can attenuate carbohydrate degradation and subsequent glucose absorption by inhibiting the catalytic activities of two digestive enzymes [15]. As presented in Figure 1, inhibition rates of αA (A) and αG (E) were calculated, thus yielding dose-response curves for BA, LI and PU across a range of concentrations. The trends were depicted in Figure 1, indicating that the three flavonoids exhibited obvious inhibitory activities toward two digestive enzymes in a dose-dependent manner. Notably, inhibition exerted by BA, LI and PU did not follow a strictly linear relationship. Within the concentration range of 0–1.2 mg/mL, inhibition rates increased substantially with rising flavonoid concentrations, whereas further increases from 1.5 to 3.0 mg/mL resulted in a tendency to stabilize. Consistent with these observations, total polyphenols extracted from buckwheat hulls on both αA and αG increased initially with the increase in polyphenol concentration, but then leveled off [19].

The half-maximal inhibitory concentration (IC_50_) serves as a pivotal parameter for evaluating the inhibitory abilities of flavonoids against αA and αG [17]. A lower IC_50_ denotes a stronger inhibitory capacity, reflecting greater efficiency of flavonoids in suppressing enzyme activity [9]. IC_50_ of BA, LI and PU for αA and αG were summarized in Table 1. Furthermore, acarbose was used as a positive control and exhibited IC_50_ of 2.039 ± 0.123 mg/mL and 3.625 ± 0.283 mg/mL against αA and αG, respectively. These values were significantly higher than those obtained for BA, LI, and PU, indicating that the three flavonoids exhibited stronger in vitro inhibitory potency toward both enzymes than acarbose under the present assay conditions. For αA, IC_50_ followed the order IC_50_ (PU) < IC_50_ (BA) < IC_50_ (LI), indicating that PU exhibited the most pronounced inhibitory effect on αA, while LI was the least effective. An identical trend was observed for αG, IC_50_ (PU) < IC_50_ (BA) < IC_50_ (LI), confirming that PU also displayed the strongest inhibition against αG. This consistent pattern might be attributable to the structural features of PU, which possessed a greater number of hydroxyl groups and double bonds compared with BA and LI. Such structural attributes likely enabled PU to engage in more extensive interactions, including hydrophobic contacts, hydrogen bonding, and van der Waals forces, with amino acid residues within the active sites of αA and αG, thereby leading to more effective suppression of enzymatic activity. Le et al. [8] reported that the IC_50_ of p-coumaric acid, quercetin, and cyanidin-3-glucoside for porcine pancreatic α-amylase was 10.4, 0.26, and 0.34, indicating that quercetin showed the strongest inhibitory effect. Furthermore, the IC_50_ of the three flavonoids for αA were significantly lower than those for αG (*p* < 0.05), which might be due to the stronger affinity between the active substances of flavonoids and αA.

### 3.2. Inhibition Kinetics of Flavonoids Against αA and αG

The inhibition kinetics of BA, LI, and PU against αA and αG were investigated using nonlinear regression and Lineweaver-Burk (LB) analysis. As shown in Figure S1, the concentrations of reducing sugar equivalents and pNP increased approximately linearly with reaction time, and the initial reaction velocities were calculated from the slopes of the corresponding linear regressions. The initial velocities were subsequently fitted to competitive, noncompetitive, and mixed inhibition models (Figure S2). The fitting results were compared using the residual sum of squares (SSE) and corrected Akaike information criterion (AICc) (Table S1). For αA, the mixed inhibition model exhibited the lowest SSE and AICc values among the three models, indicating a better fit than the competitive and noncompetitive models. In contrast, for αG, although the mixed model yielded slightly lower SSE values, the noncompetitive model showed lower AICc values, indicating that the additional parameter in the mixed model did not substantially improve the fitting performance. Thus, the preliminary model comparison favored mixed inhibition for BA, LI, and PU against αA and noncompetitive inhibition against αG.

Furthermore, LB plots were used to characterize the inhibition patterns of BA, LI, and PU against αA and αG. As shown in Figure 1B–D, LB lines for αA intersected in the second quadrant. According to Table 1, both the maximum reaction velocity (V_m_) and Michaelis constant (K_m_) decreased with increasing flavonoid concentrations, indicating a mixed inhibition pattern [18]. This suggested that BA, LI, and PU could interact with both free αA and the αA-substrate complex. Similar mixed-type inhibition of αA by bound polyphenols extracted from mung bean coat dietary fiber was reported by Sun et al. [20]. In contrast, LB lines for αG intersected near the negative *x*-axis (Figure 1F–H). With increasing flavonoid concentrations, V_m_ decreased significantly (*p* < 0.05), while Km remained relatively unchanged (*p* > 0.05), consistent with noncompetitive inhibition. These results suggested that the three flavonoids inhibited αA and αG through different kinetic mechanisms. Moreover, the K_ic_ values of BA, LI and PU against αA were consistently lower than the corresponding K_iu_ values (Table 1), suggesting a greater inhibitory affinity for free αA than for the enzyme-substrate complex. Both K_ic_ and Kiu followed the order PU < BA < LI, indicating that PU exhibited the strongest inhibitory interaction with αA. Similarly, the Kiu values for αG followed the order PU < BA < LI, suggesting a stronger inhibitory effect of PU than BA and LI. These findings were consistent with the inhibitory activity results, further indicating that the inhibitory potency of the three flavonoids against both enzymes followed the order PU > BA > LI.

### 3.3. Fluorescence Quenching Effect of Flavonoids Against αA and αG

#### 3.3.1. Intrinsic Fluorescence Analysis

**Figure 2:**
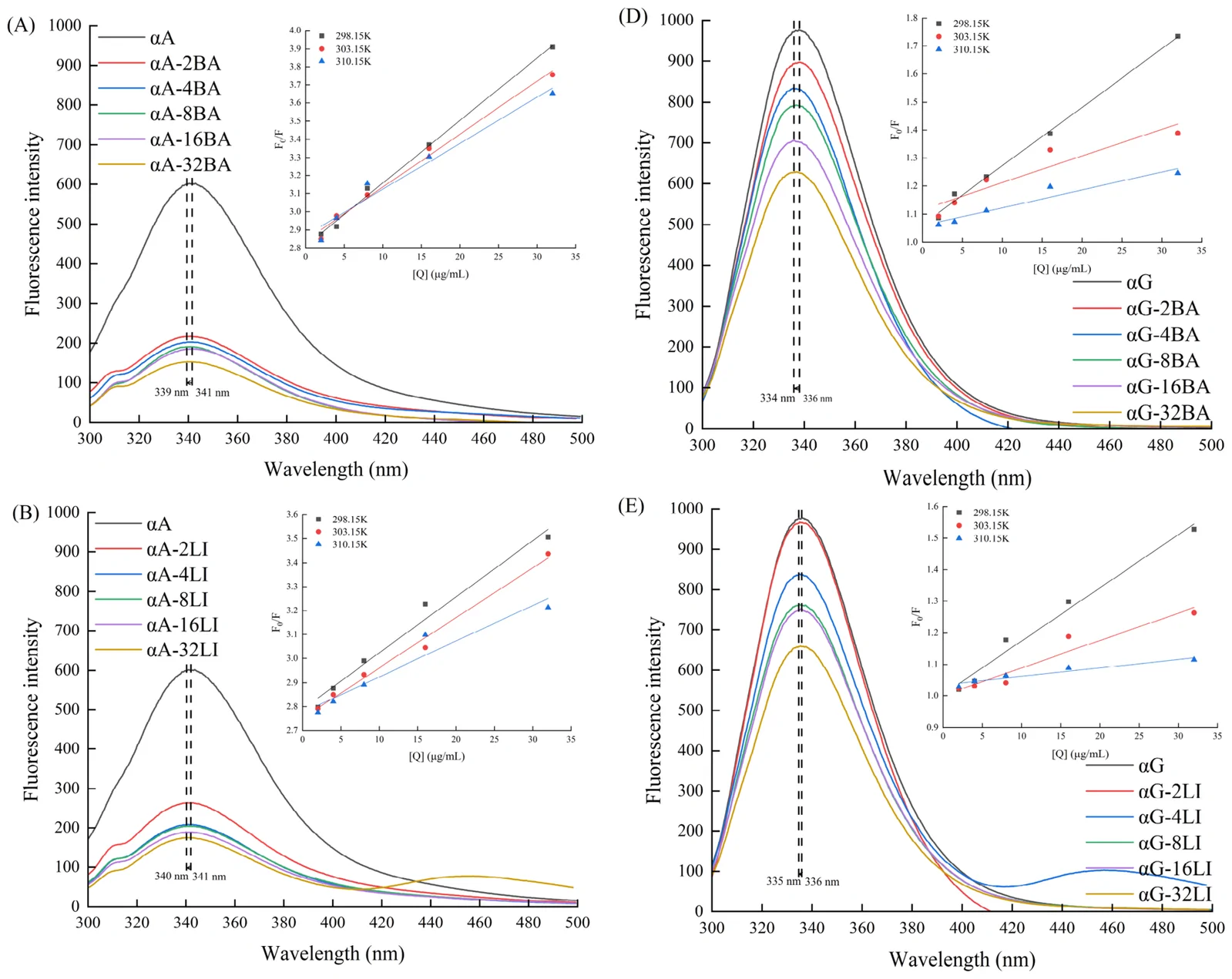

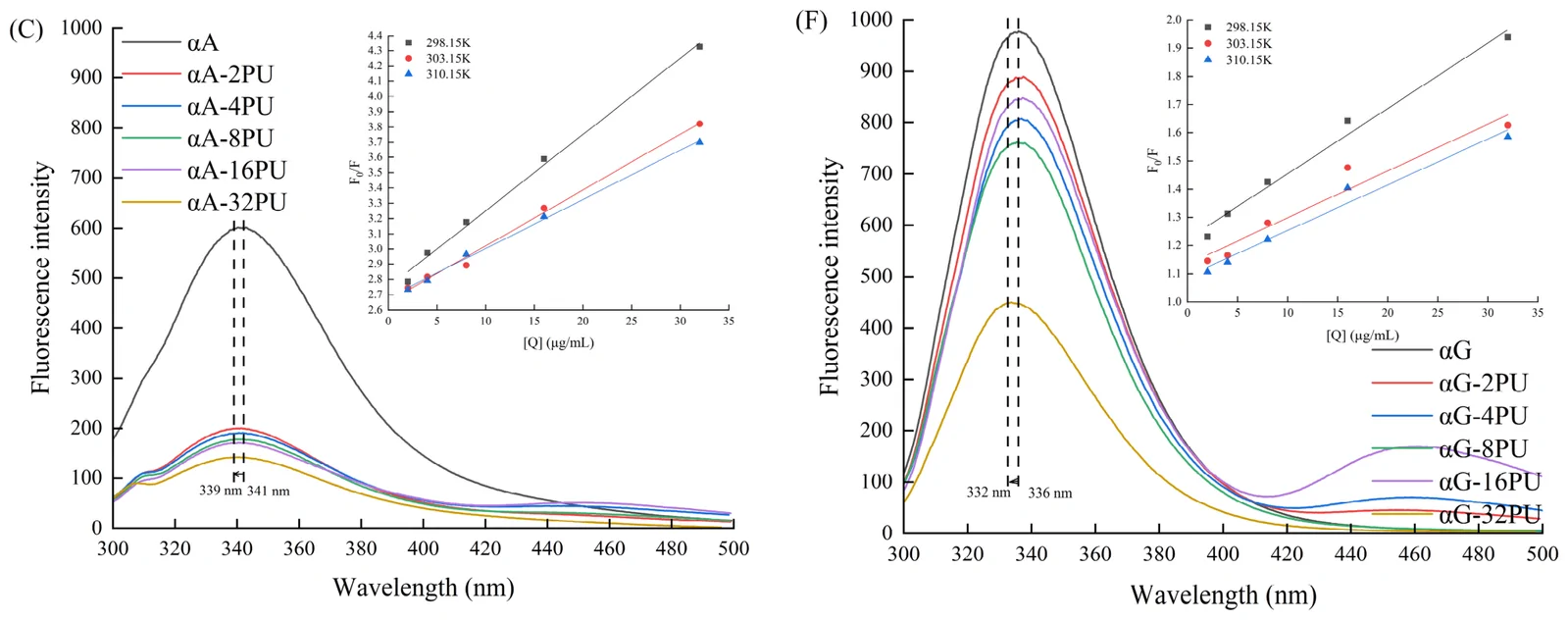
Intrinsic fluorescence spectra (insert of Stern-Volmer plots) of the effect of BA, LI and PU on αA (**A**–**C**) and αG (**D**–**F**).

The intrinsic fluorescence of αA and αG originates primarily from aromatic amino acid residues (tryptophan, tyrosine, and phenylalanine), with both intensity and emission maximum being dependent on the microenvironment surrounding these residues [21]. Figure 2 displays intrinsic fluorescence spectra of αA (A/B/C) and αG (D/E/F) in the presence of BA, LI, and PU at varying concentrations (2, 4, 8, 16, and 32 μg/mL).

As shown in Figure 2, the addition of BA, LI, and PU consistently reduced the fluorescence intensity of both αA and αG, indicating that the interaction between enzymes and flavonoids alters the polarity of the microenvironment around tryptophan residues [9], thus giving rise to fluorescence quenching. Meanwhile, intrinsic fluorescence intensity of αA and αG decreased gradually with increasing flavonoid concentrations. Furthermore, the maximum emission wavelengths of αA and αG were 341 nm and 336 nm, respectively, and all three flavonoids induced blue shifts of varying magnitude in both enzymes. This observation parallels the report by Xu et al. [19], who demonstrated that the maximum emission wavelength of αA and αG exhibited a significant blue shift after adding phenolic compounds in buckwheat hulls. As observed in Figure 2, at a flavonoid concentration of 32 μg/mL, BA (A), LI (B), and PU (C) shifted the maximum emission wavelength of αA from 341 nm to 339 nm, 340 nm, and 339 nm, respectively. This could be explained by the binding of the flavonoids to αA, which induced conformational rearrangement of the enzyme through non-covalent interactions such as hydrogen bonding and hydrophobic forces, thus driving the tryptophan residues from a relatively exposed state toward a more hydrophobic core region [22]. As shown in Figure 2, BA (D), LI (E), and PU (F) at 32 μg/mL decreased the maximum emission wavelength of αG from 336 nm to 334 nm, 335 nm, and 332 nm, respectively, indicating that their binding to αG similarly altered the tryptophan microenvironment through comparable non-covalent interactions. Compared with BA and LI, PU exhibited the strongest fluorescence quenching effect and the largest blue shift with both αA and αG. This might be attributed to the higher number of hydroxyl groups and double bonds in the molecular structure of PU, which enable it to establish more compact interactions with the enzymes, thus resulting in more pronounced conformational rearrangement and a stronger hydrophobic burial effect. Quercetin and cyanidin-3-glucoside showed a greater decline in fluorescence intensity of porcine pancreatic α-amylase than p-coumaric acid, attributed to the favorable characteristics of their conjugated structure and multiple binding sites [8].

#### 3.3.2. Fluorescence Quenching Mechanism

Fluorescence quenching analysis is commonly employed to investigate interaction mechanisms between proteins and ligands [23]. Fluorescence quenching can be classified into three types, including static quenching, dynamic quenching, and combined quenching [24].

The inset plots in Figure 2 show Stern-Volmer fitting curves of F_0_/F versus flavonoid concentration ([Q]) at 298.15 K, 303.15 K and 310.15 K. The Stern-Volmer plots for the interactions between three flavonoids and αA/αG exhibited good linear correlations, suggesting that one predominant quenching behavior occurred within the investigated concentration range. Meanwhile, Table 2 summarizes K_sv_ and K_q_ for the interactions of BA/LI/PU with αA/αG at different temperatures. As shown in Table 2, K_q_ of all samples were significantly higher than the theoretical maximum diffusion-controlled constant for dynamic quenching (2.0 × 10^10^ L⋅mol^−1^⋅s^−1^), which was consistent with a substantial static-quenching contribution associated with flavonoid-enzyme complex formation [5]. Furthermore, K_sv_ of all enzyme-flavonoid systems decreased with increasing temperature, which also supported a predominantly static quenching mechanism. Additionally, compared with BA and LI, PU exhibited the highest K_sv_, indicating its stronger interaction capability with enzymes. This phenomenon might be associated with the abundant hydroxyl groups and glycoside moiety in the PU structure, thus providing additional hydrogen-bonding sites and enhancing the stability of interactions with amino acid residues of proteins. Moreover, αA-flavonoid generally showed higher K_sv_ than αG-flavonoid, suggesting stronger binding interactions between flavonoids and αA, which might be related to the differences in the spatial structures and binding microenvironments of the two enzymes.

#### 3.3.3. Binding Constants and Binding Sites

Based on the steady-state fluorescence results, the quenching behavior of BA, LI, and PU toward αA/αG was consistent with a predominantly static quenching mechanism. Under this assumption, binding constants (K_a_) and binding sites (n) of flavonoid-enzyme complexes were calculated using the double logarithmic equation [6], and the results were summarized in Table 2. As shown in Table 2, K_a_ of all samples decreased with increasing temperature, indicating that the binding affinity between flavonoids and enzymes was gradually reduced. This suggested that the binding process was an exothermic reaction. Moreover, n values of all systems were close to 1, suggesting that BA, LI, and PU mainly interacted with αA/αG through one predominant binding site. Differences in binding affinity among flavonoids might be associated with their structural characteristics [25]. Specifically, PU contained multiple hydroxyl groups and a glycoside moiety, which could provide additional hydrogen-bonding sites and enhance non-covalent interactions with protein residues, thereby exhibiting stronger binding affinity.

#### 3.3.4. Thermodynamic Parameters

Protein-ligand interactions mainly involve four types of non-covalent forces, including hydrogen bonding, van der Waals forces, hydrophobic interactions, and electrostatic interactions [26]. Thermodynamic parameters, including enthalpy change (ΔH), entropy change (ΔS), and Gibbs free energy change (ΔG), were calculated to further elucidate the dominant interaction forces between proteins and ligands. Table 2 presents thermodynamic parameters for interactions between BA/LI/PU and αA/αG at different temperatures. As shown in Table 2, ΔG values of all systems were negative, indicating that the binding processes between the three flavonoids and αA/αG were spontaneous. Meanwhile, negative ΔH values indicated that the interactions were exothermic, whereas negative ΔS values suggested a decrease in system entropy during complex formation. According to the thermodynamic parameters, hydrogen bonding and van der Waals forces were considered the predominant driving forces involved in flavonoid-enzyme interactions.

### 3.4. Synchronous Fluorescence Analysis

**Figure 3:**
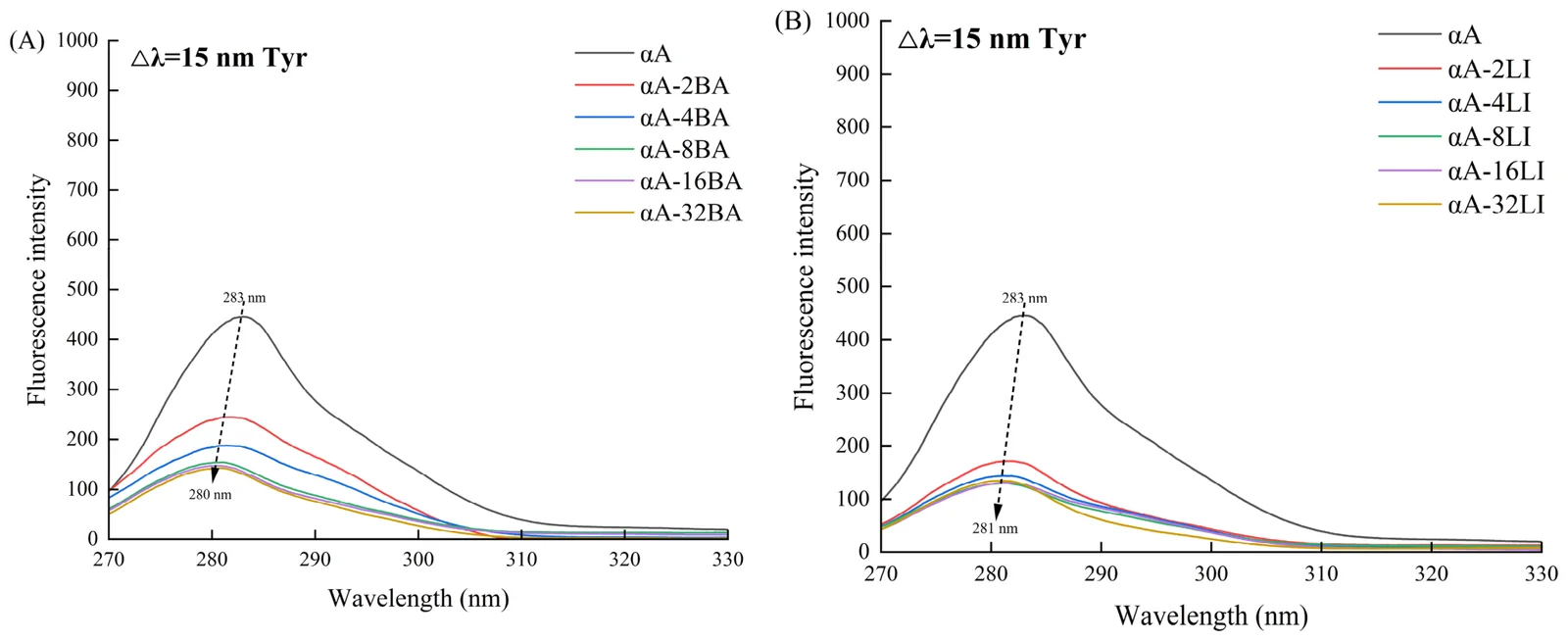

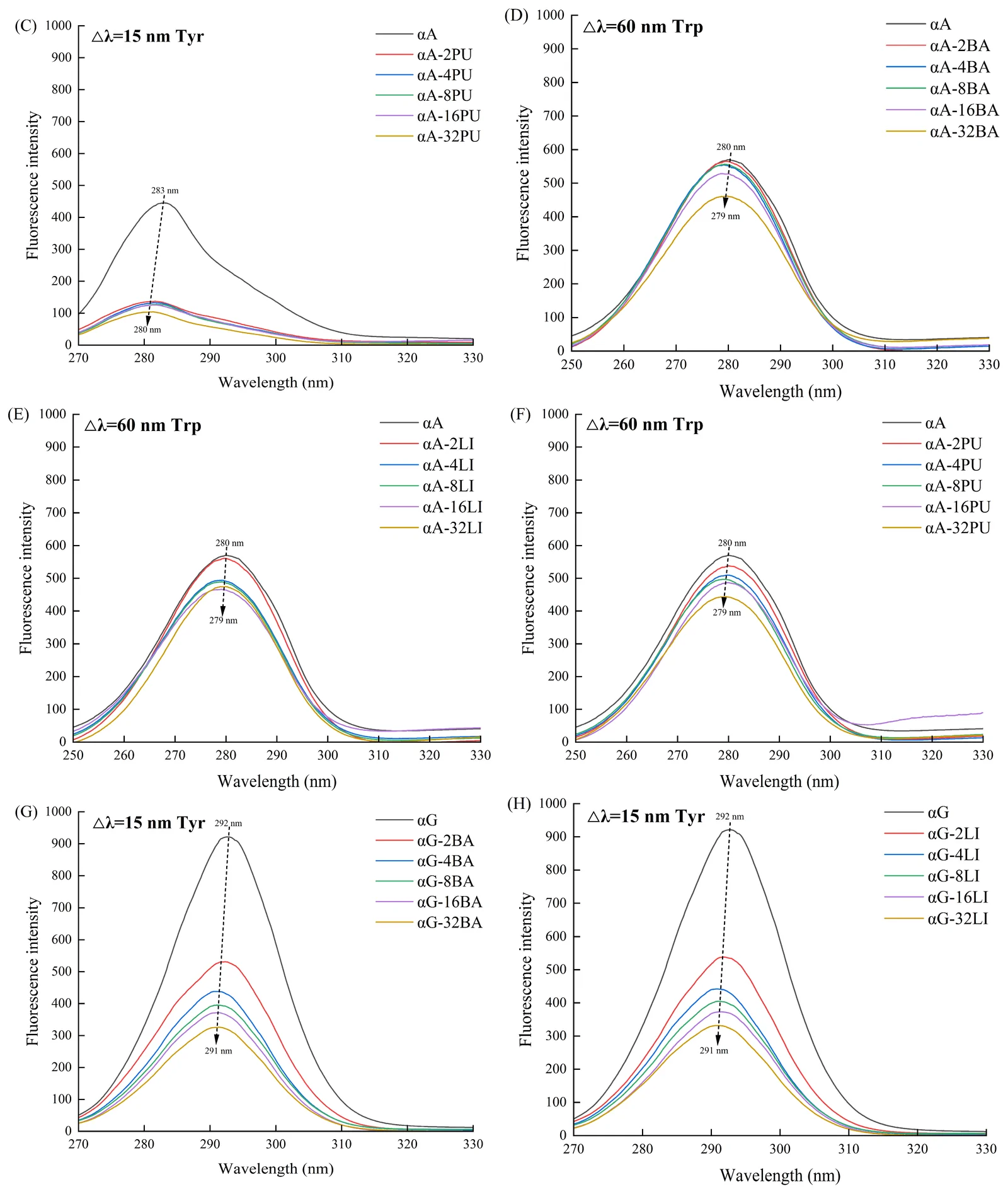

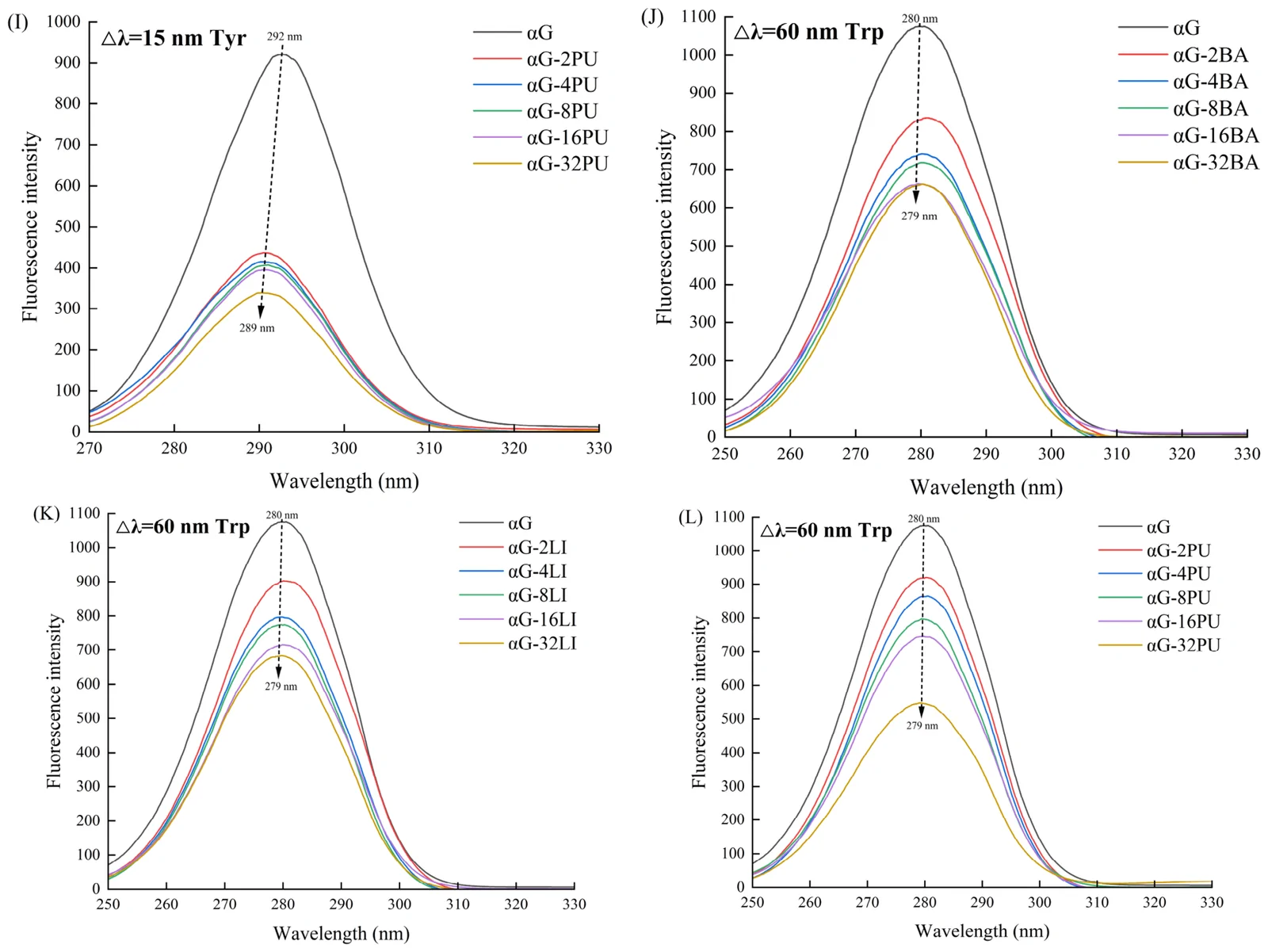
Synchronous fluorescence spectra of αA (**A**–**C**,**G**–**I**) and αG (**D**–**F**,**J**–**L**) in the presence of varying concentrations of BA, LI and PU at △λ = 15 and 60.

Synchronous fluorescence spectroscopy serves as an effective technique for evaluating alterations in the microenvironment of aromatic amino acid residues within proteins, thereby sensitively reflecting the evolutionary trend of the overall protein conformation [5]. The wavelength intervals (△λ) of 15 and 60 nm characterize the intrinsic fluorescence features of tyrosine (Tyr) and tryptophan (Trp) residues within the protein molecules, respectively [9]. Figure 3 illustrates synchronous fluorescence spectra of αA and αG at △λ = 15 nm and 60 nm in the presence of three structurally distinct flavonoids.

As depicted in Figure 3, in the synchronous fluorescence spectra at △λ = 15 nm, fluorescence intensities of Tyr residues in both αA (A/B/C) and αG (D/E/F) exhibited a progressive decline with increasing concentrations of the flavonoids, suggesting that flavonoids exerted a non-radiative energy transfer or a microenvironmental masking effect on Tyr residues inside enzyme molecules [27]. Furthermore, the introduction of flavonoids induced an obvious blue shift in the maximum emission peaks of Tyr residues. Specifically, at the flavonoid concentration of 32 μg/mL, the maximum emission wavelength of αA shifted from 283 nm to 280 nm (BA), 281 nm (LI), and 280 nm (PU), while that of αG migrated from 292 nm to 291 nm (BA), 291 nm (LI), and 289 nm (PU), respectively. This blue-shifting phenomenon revealed a reduction in the polarity and a concomitant increase in the hydrophobicity of the microenvironment harboring Tyr residues. The above results were consistent with those of Liu et al. [9], which found that Lonicera caerulea berry polyphenol-wheat starch caused a blue shift of 1 nm in the fluorescence peak of Tyr residues of αA. This could be attributed to the fact that the binding of flavonoids to enzymes induced a conformational rearrangement of the polypeptide segments. It induced a conformational rearrangement of its polypeptide chain segments, thus causing Tyr residues that were originally partially exposed on the molecular surface to be embedded or aggregated into the more hydrophobic core region of proteins [28]. Concurrently, as shown in Figure 3, in the synchronous fluorescence spectra at △λ = 60 nm, fluorescence intensities of Trp residues in αA (G/H/I) and αG (J/K/L) generally displayed a progressive downward trend as the flavonoid concentration increased. This further confirmed the existence of robust non-covalent interactions between ligands and enzyme molecules, which effectively quenched the intrinsic fluorescence of Trp residues. Moreover, the incorporation of flavonoids also caused a slight blue shift in the maximum emission peaks of both αA and αG. This marginal shift indicated that the polarity of the microenvironment surrounding Trp residues underwent only a relatively mild adjustment during the complexation process, transitioning toward a marginally enhanced hydrophobic environment [29]. These results collectively demonstrated that three structurally distinct flavonoids drove the conformational rearrangement of αA and αG by simultaneously altering the microenvironments of both Tyr and Trp residues, thereby hindering the highly efficient binding of substrates to the active sites of enzymes. Polyphenols extracted from mung bean coat dietary fiber combined with αA, which led to a change in the hydrophobic microenvironment around the tryptophan residue [30]. This might be one of the triggering factors for the inhibition of αA by polyphenols. Additionally, the fluorescence quenching effect on Trp residues of αA was more pronounced than that on Tyr residues, further implying that the binding sites of flavonoids on αA were located in closer proximity to the Trp residues. Conversely, the fluorescence quenching of Tyr residues in αG outperformed that of Trp residues, which further substantiated that the binding sites of flavonoids on αG were situated closer to Tyr residues.

### 3.5. UV-Vis Spectroscopy Analysis

**Figure 4:**
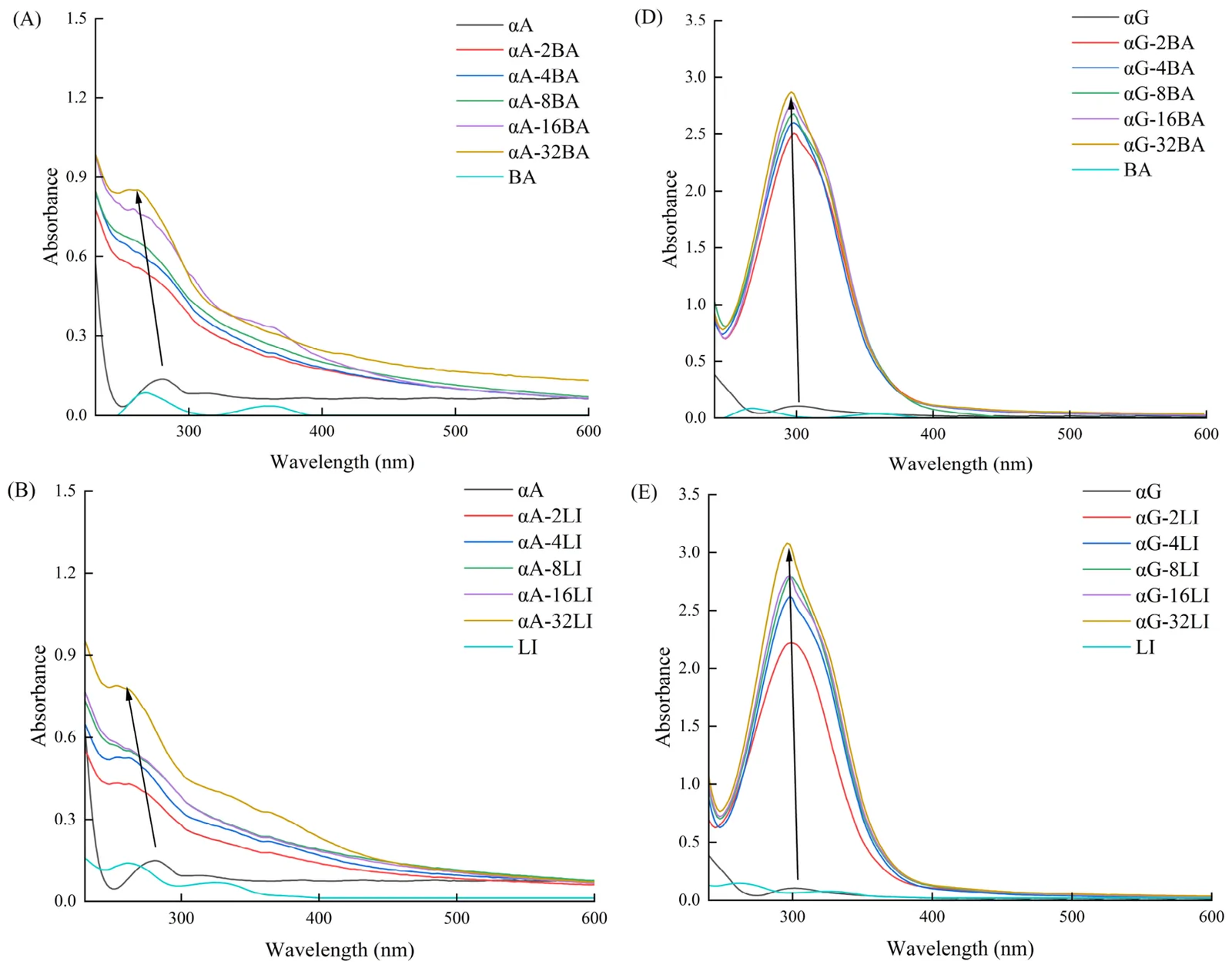

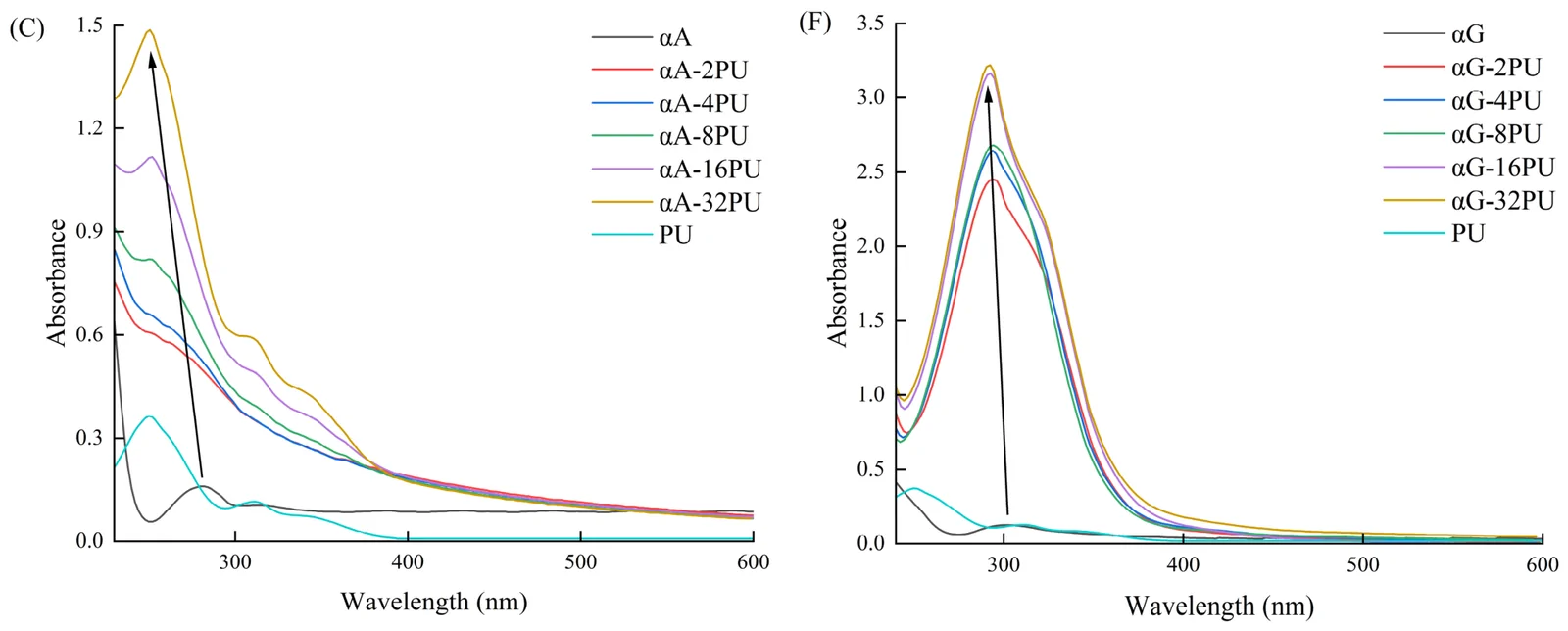
Effect of different concentrations of BA, LI and PU on UV-Vis spectra of αA (**A**–**C**) and αG (**D**–**F**).

UV-Vis spectroscopy serves as a valuable technique for probing changes in the polarity of the protein microenvironment and electronic transitions upon enzyme-ligand interactions, with the position and intensity of protein absorption peaks reflecting conformational alterations [17]. Figure 4 presents UV-Vis spectra of αA (A/B/C) and αG (D/E/F) in the presence of BA, LI, and PU at various concentrations (2, 4, 8, 16, and 32 μg/mL).

As shown in Figure 4, αA and αG exhibited characteristic peaks near 280 nm and 300 nm, respectively, which were primarily associated with electronic transitions of aromatic amino acid residues (tryptophan, tyrosine, and phenylalanine). This was consistent with the research results of the previous study [19]. UV absorption of these residues originated from the conjugated π-electron systems in their side chains, enabling light absorption within specific wavelength ranges [30]. With increasing concentrations of BA, LI, and PU, the absorption intensities of αA and αG gradually increased, indicating that flavonoid addition altered the UV absorption behavior of the enzyme systems. Among the three flavonoids, PU induced the most pronounced enhancement in absorbance intensity, followed by LI and BA, suggesting stronger interactions between PU and the enzymes, which was consistent with its higher inhibitory activity and binding affinity. For instance, UV-Vis absorbance of α-amylase rose with increasing phenolic acid (hydroxybenzoic acid and ferulic acid) concentrations [10]. It should be noted that BA, LI, and PU possess intrinsic UV absorption in the investigated wavelength range. Therefore, the spectral changes observed in the enzyme-flavonoid systems may arise from the combined contributions of flavonoid absorption and alterations in the local environment of protein chromophores. Accordingly, the observed changes were interpreted as evidence of flavonoid-enzyme interactions rather than being solely attributed to conformational alterations of the enzymes. A comparable result was documented by Xu et al. [19], who found that UV absorption wavelengths of αG shifted from 300 nm to 296 nm after adding buckwheat hull polyphenols, and the characteristic peak exhibited a slight blue shift as the concentration of polyphenols increased. However, although the binding of three flavonoids to αG also altered the microenvironment of aromatic residues, slightly enhancing hydrophobicity, the extent of conformational perturbation in αG appeared weaker than that in αA. This difference might be related to the distinct structural features of the active sites of the two enzymes and different binding modes of flavonoids [6]. Moreover, compared with BA and LI, PU induced the most pronounced blue shift in the characteristic UV absorption peaks of both αA and αG, suggesting that PU binding triggered more substantial conformational changes, thus leading to tighter burial of aromatic residues [31]. This observation was consistent with the strongest inhibitory capacity of PU demonstrated in the activity and kinetic experiments.

### 3.6. Circular Dichroism Spectroscopy Analysis

**Figure 5:**
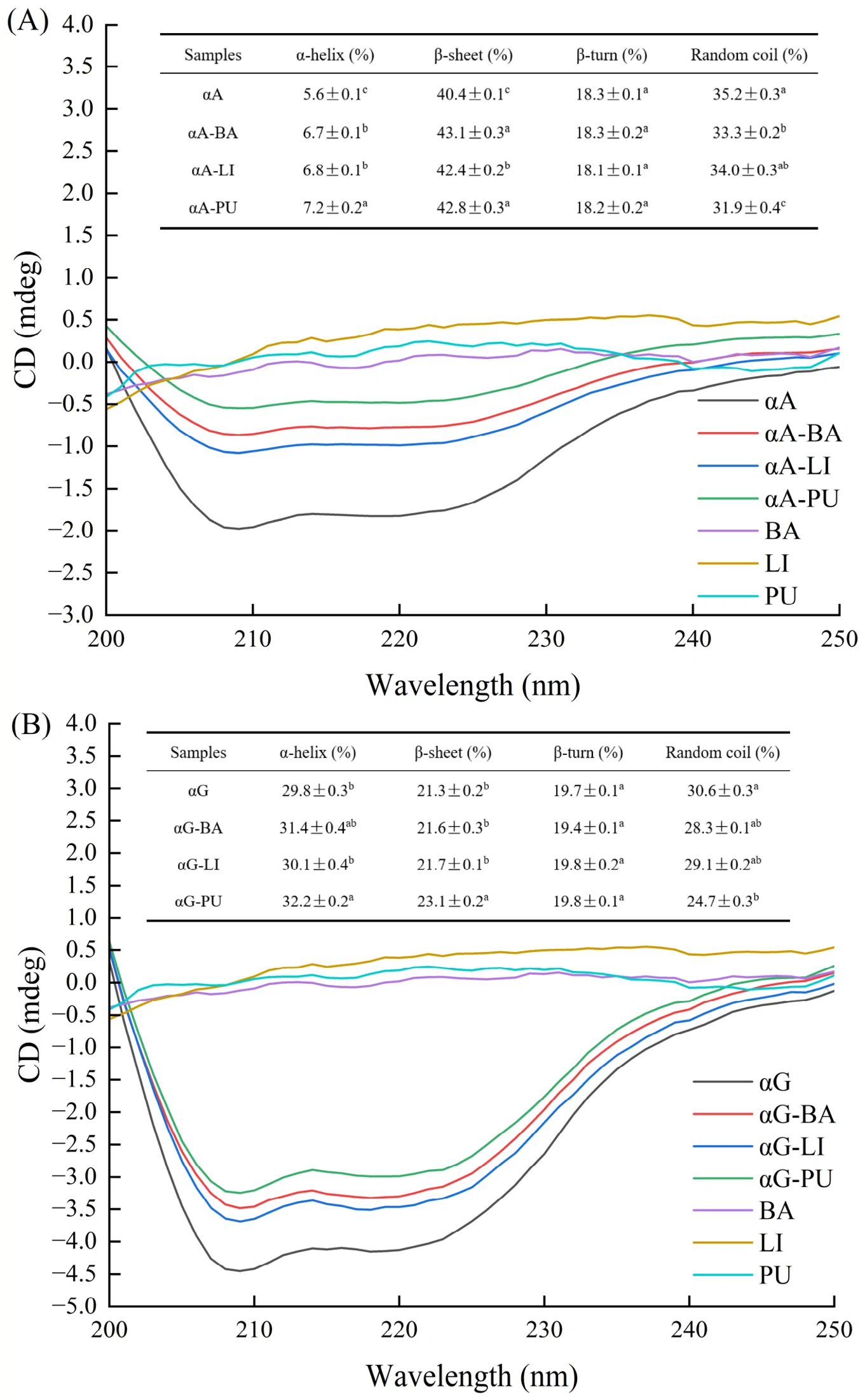

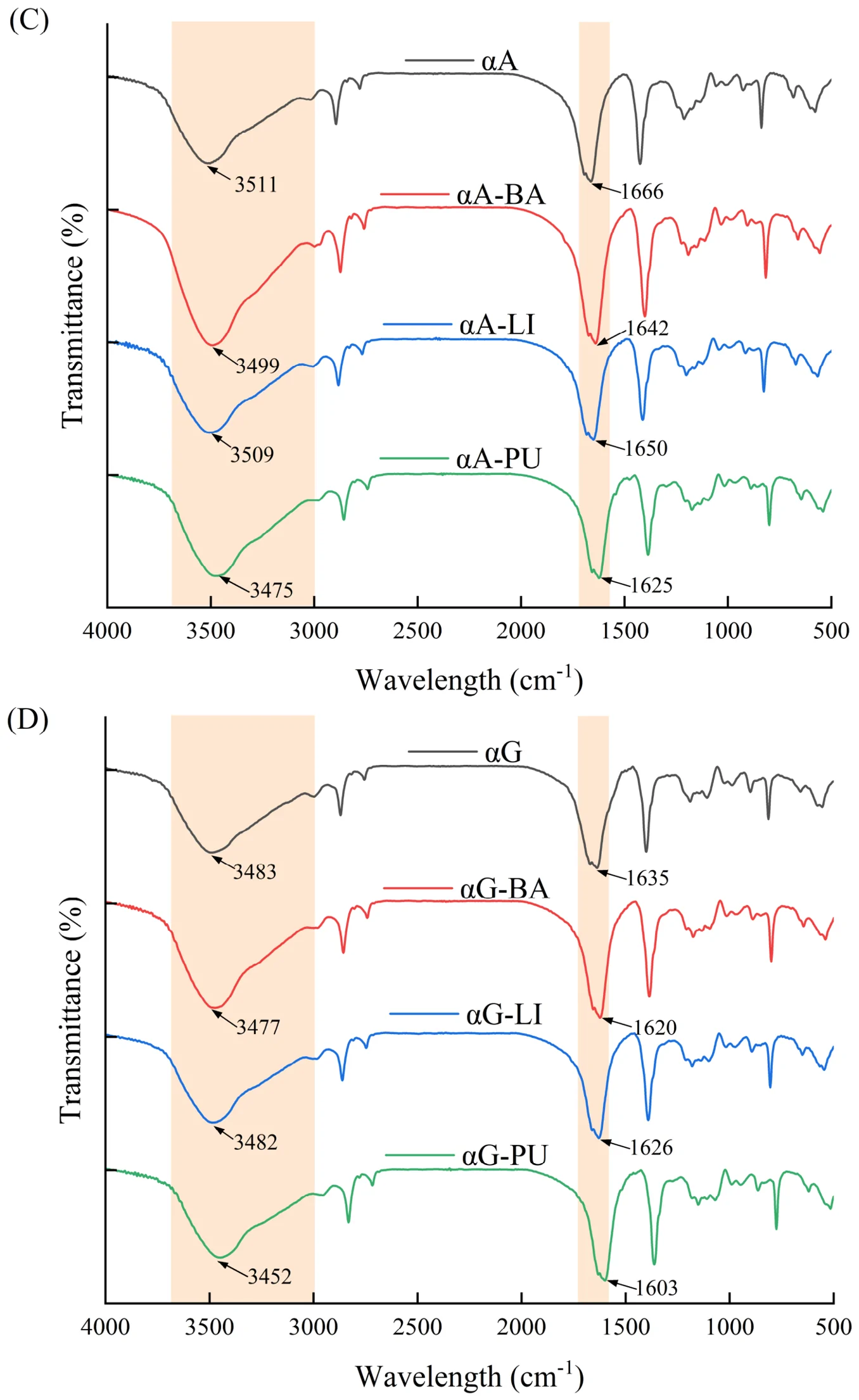

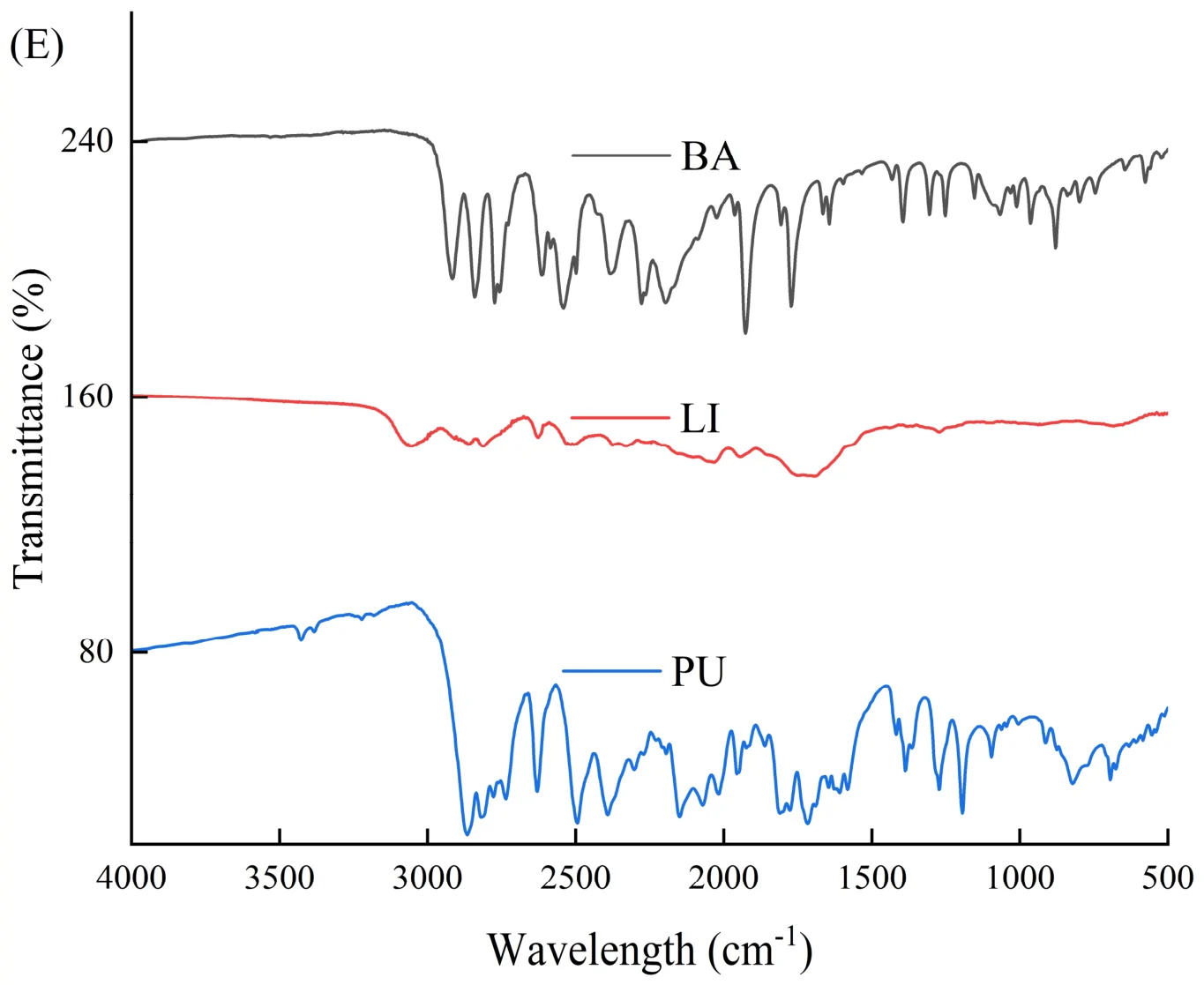
Circular dichroism spectra and secondary structure contents (insets) of αA (**A**) and αG (**B**) in the absence and presence of BA, LI or PU (8 μg/mL). FTIR spectra of αA (**C**), αG (**D**), and BA, LI and PU (**E**). Different lowercase superscript letters within the same column indicate significant differences (*p* < 0.05), whereas identical or shared letters indicate no significant difference. The orange-shaded areas indicate the O–H/N–H stretching region (3700–3000 cm^−1^) and the amide I region (1700–1600 cm^−1^).

Circular dichroism (CD) spectroscopy has been widely employed to investigate protein conformation by monitoring changes in secondary structure content, with the far-UV region reflecting the backbone conformation [4]. Figure 5 displays CD spectra of αA (A) and αG (B) in the presence of BA, LI, and PU at a concentration of 8 μg/mL. Both digesting enzymes exhibited characteristic α-helical peaks near 208 and 220 nm and a typical β-sheet peak around 215–217 nm. The addition of three flavonoids reduced the negative ellipticity of CD spectroscopy, indicating alterations in the secondary structures of αA and αG.

Secondary structure compositions of native αA and αG and their complexes with BA, LI, and PU were estimated using the CONTIN method for quantitative analysis, shown in Figure 5. Specifically, native αA contained 5.6% α-helix, 40.4% β-sheet, and 18.3% β-turn. Native αG contained 29.8% α-helix, 21.3% β-sheet, and 19.7% β-turn. The addition of BA, LI, and PU significantly increased α-helix and β-sheet contents of both enzymes (*p* < 0.05), while significantly decreasing random coil content (*p* < 0.05), whereas β-turn content remained essentially unchanged (*p* > 0.05). Therefore, BA, LI, and PU all promoted the conversion of secondary structure from random coil to α-helix and β-sheet in both αA and αG. For instance, compared to αA, α-helix and β-sheet of αA-PU increased by 32.0% and 5.9%, while random coil of αA-PU decreased by 10.3%. However, compared to αG, the α-helix and β-sheet of αG-PU increased by 8.1% and 8.5%, while the random coil of αG-PU decreased by 23.9%. α-helical and β-sheet structures were primarily stabilized by hydrogen bonds; the increase in these two ordered elements, together with the decrease in random coil content, was conducive to enzyme stabilization. This phenomenon might result from the insertion of flavonoids into the hydrophobic surface of the enzymes, disrupting certain regions and inducing rearrangement of chain segments [5]. Therefore, enzyme-flavonoid complexes appeared to be formed through non-covalent interactions, mainly hydrogen bonding and hydrophobic forces. Consistent with the present findings, the addition of spherical nanocrystalline cellulose significantly reduced α-helix content, significantly increased β-turns and β-folds content of αA, thereby altering the secondary structure of the enzyme [32]. Furthermore, compared with enzyme-BA and enzyme-LI, enzyme-PU complexes exhibited higher α-helix and β-sheet contents, indicating that PU formed a more compact complex system with the enzymes and that this interaction is associated with stronger hydrophobicity. As shown in Figure 5, the addition of flavonoids disrupted the native conformation of enzymes, thereby impeding the formation of the active site, preventing substrate binding, and ultimately compromising enzymatic activity [30].

### 3.7. Fourier Transform-Infrared Spectroscopy Analysis

Fourier transform-infrared (FT-IR) spectroscopy can be used to monitor secondary structure changes of proteins after binding a ligand [33]. Therefore, in this study, FTIR spectroscopy was employed to further investigate the effects of BA, LI, and PU on the secondary structures of αA and αG, as well as the intermolecular interactions involved. Figure 5 displays FTIR spectra of αA (C) and αG (D) at BA, LI, and PU concentrations of 8 μg/mL.

FT-IR spectra of BA, LI, and PU are shown in Figure 5E. All three flavonoids exhibited characteristic absorption bands associated with their hydroxyl, carbonyl, and aromatic structures. BA showed characteristic peaks at approximately 3320 cm^−1^, 1650 cm^−1^, and 1605–1450 cm^−1^, which were mainly attributed to O-H stretching vibration, C=O stretching vibration, and C=C stretching vibrations of the aromatic rings, respectively. LI displayed typical absorption bands around 3400 cm^−1^, 1630 cm^−1^, and 1510–1450 cm^−1^, corresponding to hydroxyl groups, carbonyl groups, and aromatic skeletal vibrations. PU exhibited characteristic peaks near 3400 cm^−1^, 1655 cm^−1^, 1600 cm^−1^, and 1515 cm^−1^, which were assigned to O-H stretching, C=O stretching, and aromatic ring vibrations, respectively. As shown in Figure 5, no new absorption peaks appeared after the interaction between flavonoids and αA/αG, indicating that no new covalent bonds were formed. Therefore, the interaction between flavonoids and digestive enzymes was mainly mediated by non-covalent interactions. The vibrations in the regions of 1700–1600 cm^−1^ and 1600–1500 cm^−1^ corresponded to amide I and amide II bands of proteins, which arose from C=O and C-N stretching vibrations, respectively [34]. Among these regions, the amide I band is particularly sensitive to changes in the protein microenvironment and was therefore used to evaluate conformational alterations induced by flavonoid binding. In the 1700–1600 cm^−1^ region, the maximum absorption peaks of αA and αG were observed at 1666 cm^−1^ and 1635 cm^−1^. The addition of three flavonoids caused a red shift in the maximum absorption peaks of digestive enzymes in this region. For example, the maximum absorption peaks of αA-BA, αA-LI and αA-PU were 1642 cm^−1^, 1650 cm^−1^ and 1625 cm^−1^, respectively, while those of αG-BA, αG-LI and αG-PU were 1620 cm^−1^, 1626 cm^−1^ and 1603 cm^−1^, respectively. These spectral shifts suggested that flavonoid binding altered the local environment surrounding peptide bonds, which may be associated with hydrogen bonding and other non-covalent interactions between flavonoids and enzymes [6]. The addition of catechol, quercetin, and hesperidin caused a significant red shift in the infrared characteristic vibrations of αA [35]. Furthermore, compared with BA and LI, PU induced the most pronounced red shifts in both C=O and N-H absorption peaks of αA and αG. This might be explained by the fact that PU possessed more hydroxyl groups and double bonds in its molecular structure [36], thereby enabling it to form a denser hydrogen-bonding network and stronger hydrophobic interactions with αA and αG. This result was consistent with the circular dichroism spectroscopy findings. It further confirmed that PU had the strongest binding with the two digestive enzymes, thereby exerting the most significant inhibitory effect on αA and αG.

### 3.8. Molecular Docking

**Figure 6:**
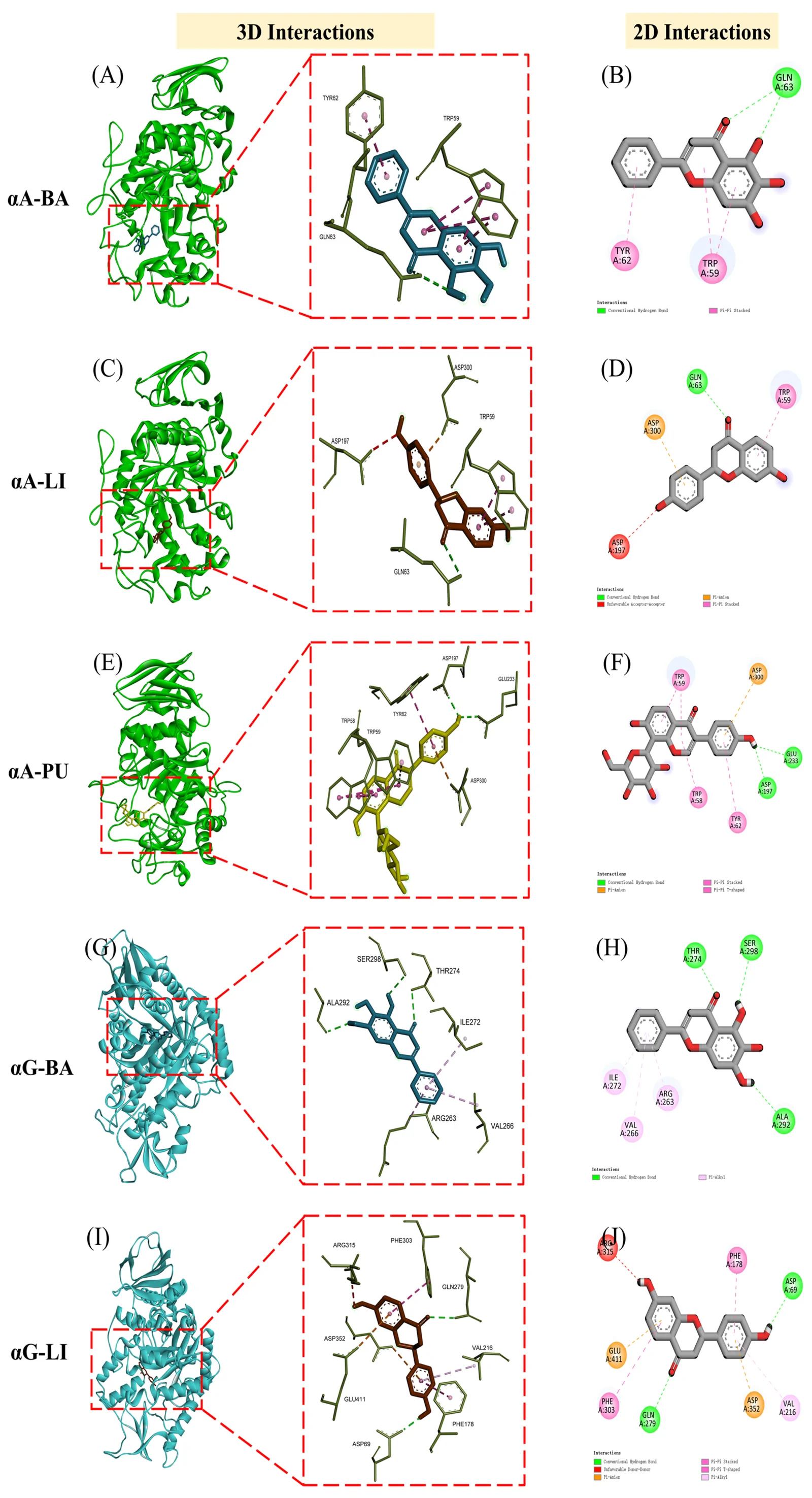

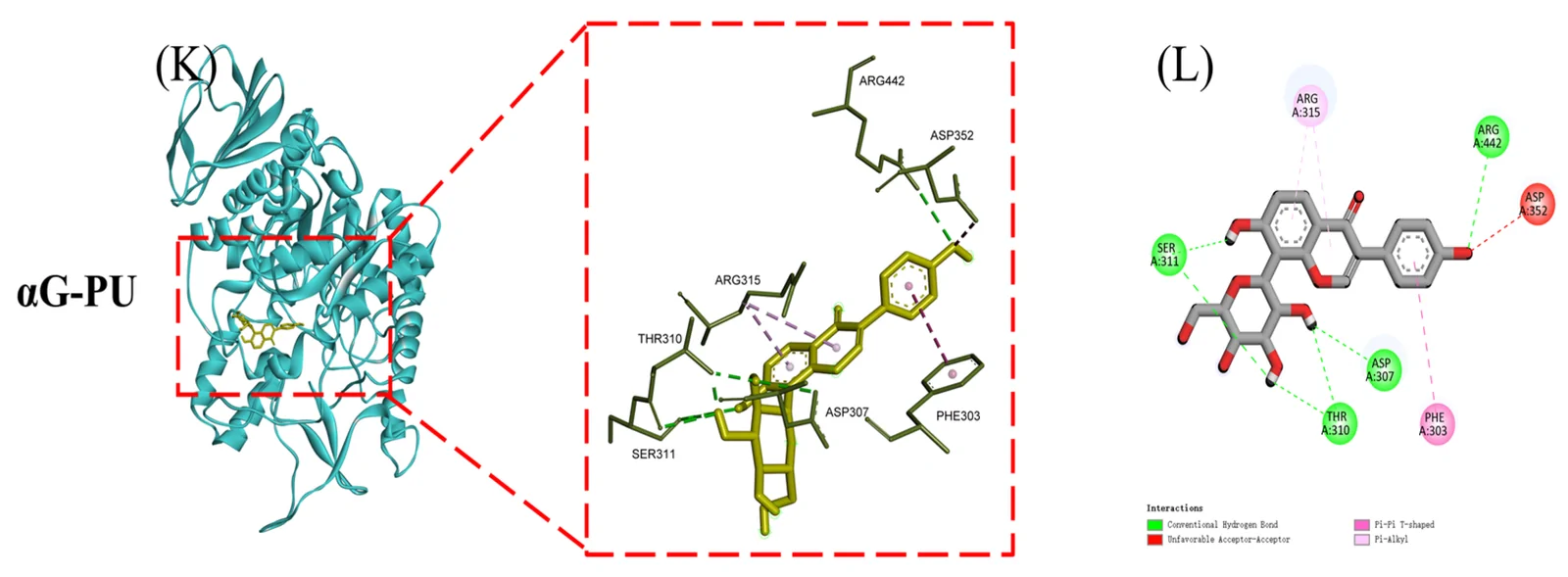
The three-dimensional interaction schematic diagram of molecular docking results between BA (**A**,**G**), LI (**C**,**I**), and PU (**E**,**K**) with αA/αG. The two-dimensional interaction schematic diagram of molecular docking results between BA (**B**,**H**), LI (**D**,**J**), and PU (**F**,**L**) with αA/αG.

Molecular docking is a technique that uses computer simulation to identify and predict the structure of ligand-receptor complexes, thus being recognized as an important approach for exploring interaction modes [9]. Table 3 presents the binding energy (E_b_) results for three flavonoids with different structures against αA and αG. As shown in Table 3, the absolute E_b_ values of the three flavonoids against both αA and αG followed the same order: PU > LI > BA. These results indicate that PU possesses the strongest affinity for forming a more stable conformation with the enzymes. Furthermore, Figure 6 displays three-dimensional and two-dimensional schematic diagrams of the molecular docking results of BA, LI, and PU with αA/αG.

As shown in Figure 6, BA (A), LI (C), and PU (E) successfully embedded themselves into the active cavity of αA, thereby forming multiple non-covalent interactions with key catalytic residues within the cavity. The three critical active-site residues of αA are Asp 197, Glu 233, and Asp 300 [37]. As shown in Figure 6D,F, hydroxyl groups of LI and PU interacted via hydrogen bonds with the carbonyl groups on the catalytic residues at the active center of αA, with these hydrogen bonds involving Asp 197 (LI), Glu 233, and Asp 197 (PU), respectively. Thus, the binding of LI and PU to αA exhibited a mixed-type inhibition, which was consistent with the results of enzyme kinetic experiments. Additionally, as shown in Figure 6B, BA formed an additional hydrogen bond with Gln 63 around the active site. Therefore, hydrogen bonds were the predominant interaction force between the three flavonoids and αA.

As exhibited in Figure 6, none of the three flavonoids directly interacted with the active site of αG. In contrast to αA, they bound to the peripheral region of the active pocket composed of hydrophobic amino acids and polar residues. The key residues of αG involved in substrate catalysis and binding were Ala537, Thr533, and Ser557 [19]. As shown in Figure 6H, BA was mainly connected to αG via hydrogen bonds and π-alkyl interactions. As exhibited in Figure 6J, LI interacted with αG primarily through hydrogen bonds, π-alkyl interactions, donor-donor repulsion, and π-π stacking. As shown in Figure 6L, PU exhibited the most extensive interaction network, binding to αG mainly through hydrogen bonds, receptor-receptor interactions, π-alkyl, and π-π T-shaped interactions. Therefore, hydrogen bonds were the predominant interaction force between the three flavonoids and αG. Moreover, because the binding sites of all three flavonoids were located outside the substrate catalytic center, they did not hinder the entry of substrate molecules into the active pocket, thus manifesting as non-competitive inhibition, which was consistent with enzyme kinetic results.

To further assess the reliability of the predicted ligand conformations, an independent redocking analysis was performed using the co-crystallized α-D-glucose (GLC) ligand of Saccharomyces cerevisiae isomaltase (PDB ID: 3A4A), which was used as the structural model for αG. The redocked GLC exhibited a docking score of −6.741 kcal/mol. The heavy-atom root-mean-square deviation (RMSD) between the redocked and crystallographic conformations was 0.478 Å in the fixed protein coordinate frame and 0.188 Å after ligand superposition. Both values were below the commonly accepted reference threshold of 2.0 Å, indicating close agreement between the predicted and crystallographic GLC conformations. In contrast, the crystal structure of αA (PDB ID: 5KEZ) contains a co-crystallized peptide inhibitor rather than a conventional small-molecule ligand, and therefore was not subjected to the same small-molecule redocking procedure. This Glide-based redocking analysis provided an independent assessment of ligand-pose recovery for 3A4A. Furthermore, the quantity and position of -OH groups are crucial for the formation of strong hydrogen bonds [38]. The binding affinity of polyphenols to digestive enzymes is positively correlated with the number of hydrogen bonds formed in the complex [8]. Compared with BA and LI, PU bound more hydrogen bonds with αA (3) and αG (4), thereby exhibiting a stronger binding affinity with digestive enzymes.

### 3.9. Molecular Dynamics Simulation

**Figure 7:**
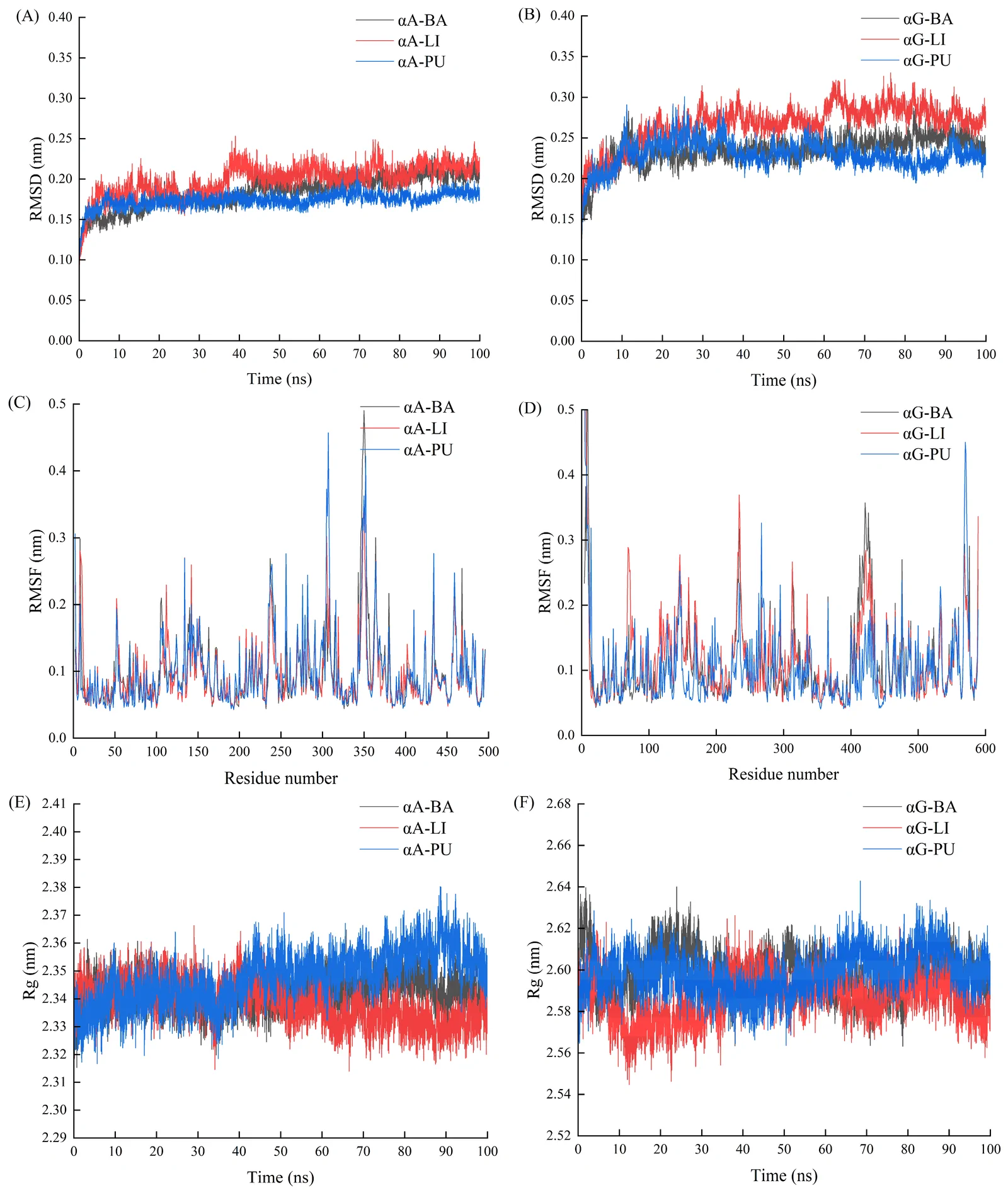

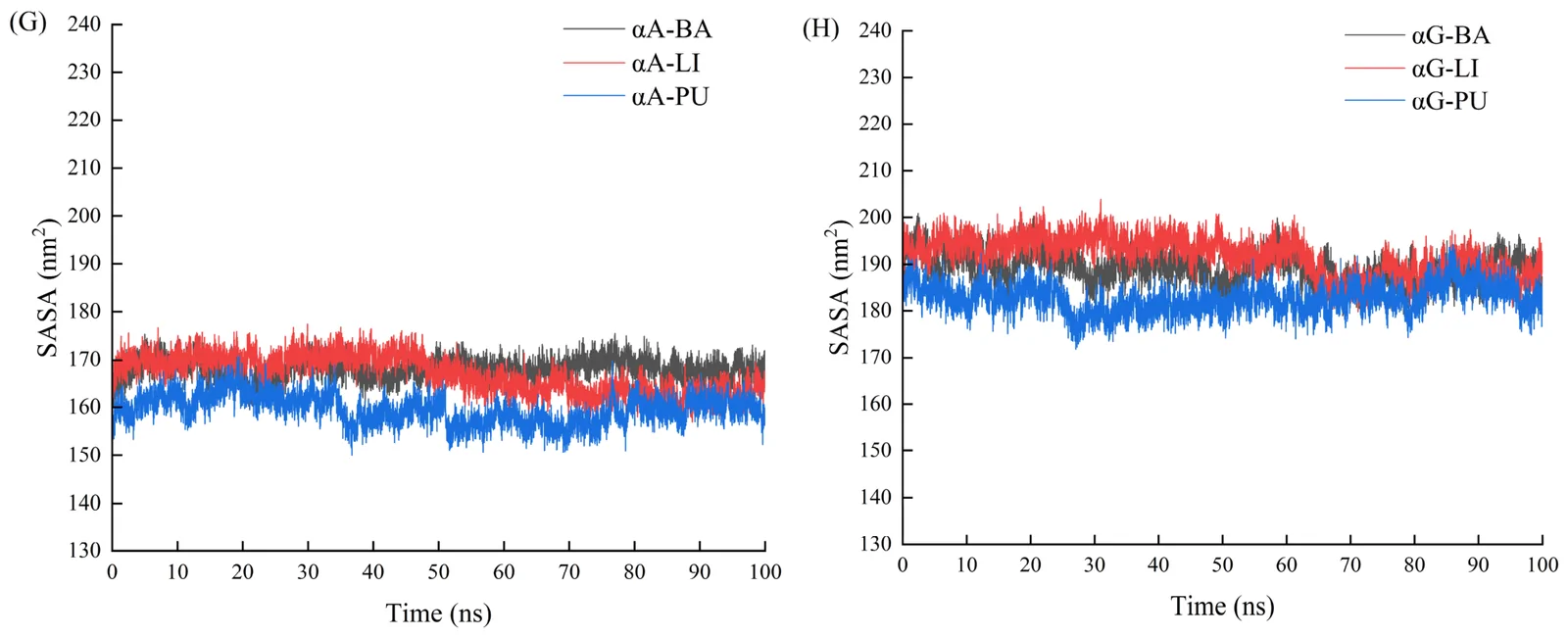
The changes in RMSD (**A**,**B**), RMSF (**C**,**D**), Rg (**E**,**F**) and SASA (**G**,**H**) of the complexes formed by three flavonoids with αA/αG during molecular dynamics simulation.

Molecular dynamics (MD) simulation can provide an atomistic-level description of the dynamic behavior of protein-ligand complexes over time, further elucidating the effects of ligand binding on protein conformational stability and structural alterations [39]. Figure 7 presents the dynamic structural changes of αA/αG with BA/LI/PU during a 100 ns MD simulation, including root mean square deviation (RMSD), root mean square fluctuation (RMSF), radius of gyration (Rg), and solvent accessible surface area (SASA).

RMSD was used to evaluate conformational stability and the equilibrium state of the protein during MD simulation [40]. As shown in Figure 7, RMSD of αA-flavonoids (A) and αG-flavonoids (B) increased slightly during the initial 20 ns and subsequently reached a relatively stable state, indicating that all systems underwent an initial conformational adjustment and gradually achieved dynamic equilibrium. Therefore, it could be inferred that at the end of the MD simulation, both systems had reached equilibrium [41]. Compared with BA and LI, αA-PU and αG-PU exhibited lower RMSD with smaller fluctuations throughout the simulation period, suggesting that PU could maintain a more stable enzyme conformation. Furthermore, compared with αG-flavonoid, αA-flavonoid showed more stable RMSD profiles, indicating a stronger conformational stability of αA upon flavonoid binding.

RMSF was performed to evaluate the local flexibility and residue-level fluctuations of proteins during MD simulation [42]. As shown in Figure 7, most amino acid residues of αA-flavonoids (C) and αG-flavonoids (D) exhibited relatively low RMSF values, suggesting that flavonoids did not induce substantial global structural fluctuations of enzymes [43]. RMSF values of αA-flavonoid remained below 0.1 nm overall, with obvious fluctuation peaks observed mainly in the regions of residues 200–250, 350–380, and 450–480. This indicated that BA, LI, and PU could reduce the flexibility of individual residues in αA, contributing to the formation of stable enzyme-ligand complexes. However, the effects of flavonoids on αG residue mobility were more pronounced. For αG-flavonoid, significant RMSF peaks exceeding 0.2 nm were observed around residue regions 210–240 and 350–370, indicating that flavonoid binding induced enhanced flexibility in specific regions of αG.

Rg was used to characterize the compactness and overall structural integrity of proteins during MD simulation [44]. Figure 7 exhibits the trend of Rg of αA-flavonoids (E) and αG-flavonoids (F). Rg of αA-flavonoid remained relatively stable throughout the 100 ns simulation, mainly ranging from 2.31 to 2.35 nm, without obvious structural expansion [45]. In contrast, the incorporation of flavonoids resulted in a gradual increase in the Rg values of αG complexes during the simulation, indicating more pronounced conformational adjustments of αG. Moreover, compared with BA and LI, enzyme-PU complexes exhibited relatively lower variations in Rg, suggesting that PU was more effective in maintaining a stable and compact enzyme structure.

SASA was performed to investigate the solvent exposure of protein-ligand complexes and evaluate the conformational changes associated with ligand binding [46]. Figure 7 shows the trend of SASA of αA-flavonoids (G) and αG-flavonoids (H). SASA of αA-flavonoid remained relatively stable during the 100 ns simulation, mainly ranging from 158 to 178 nm^2^. However, SASA of αG-flavonoid complexes increased during the initial simulation stage and remained at a relatively high level in the later stage [47]. Furthermore, PU binding exhibited a favorable effect on maintaining enzyme conformational stability, whereas LI binding may induce more pronounced structural expansion or increased surface exposure of the enzyme.

**Figure 8:**
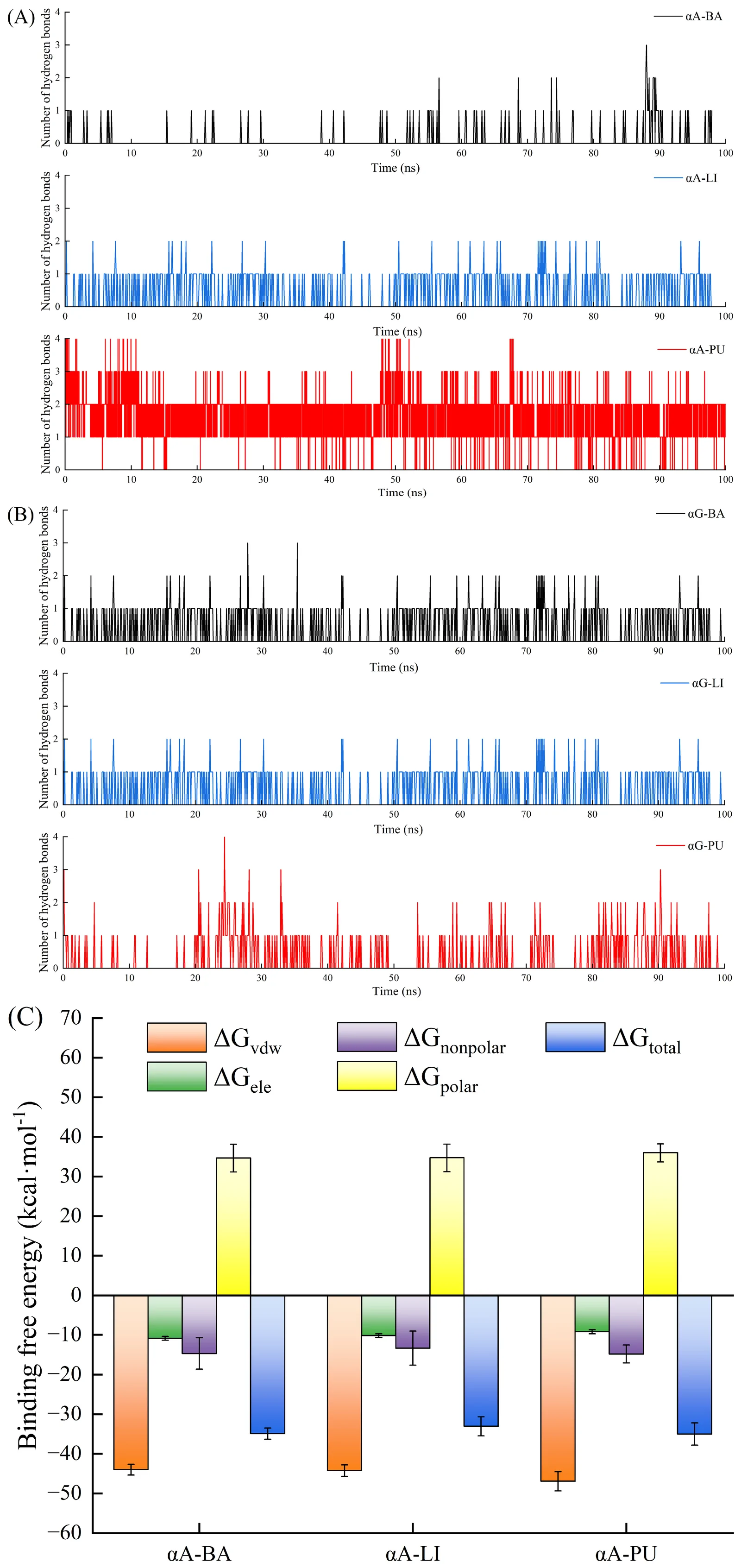

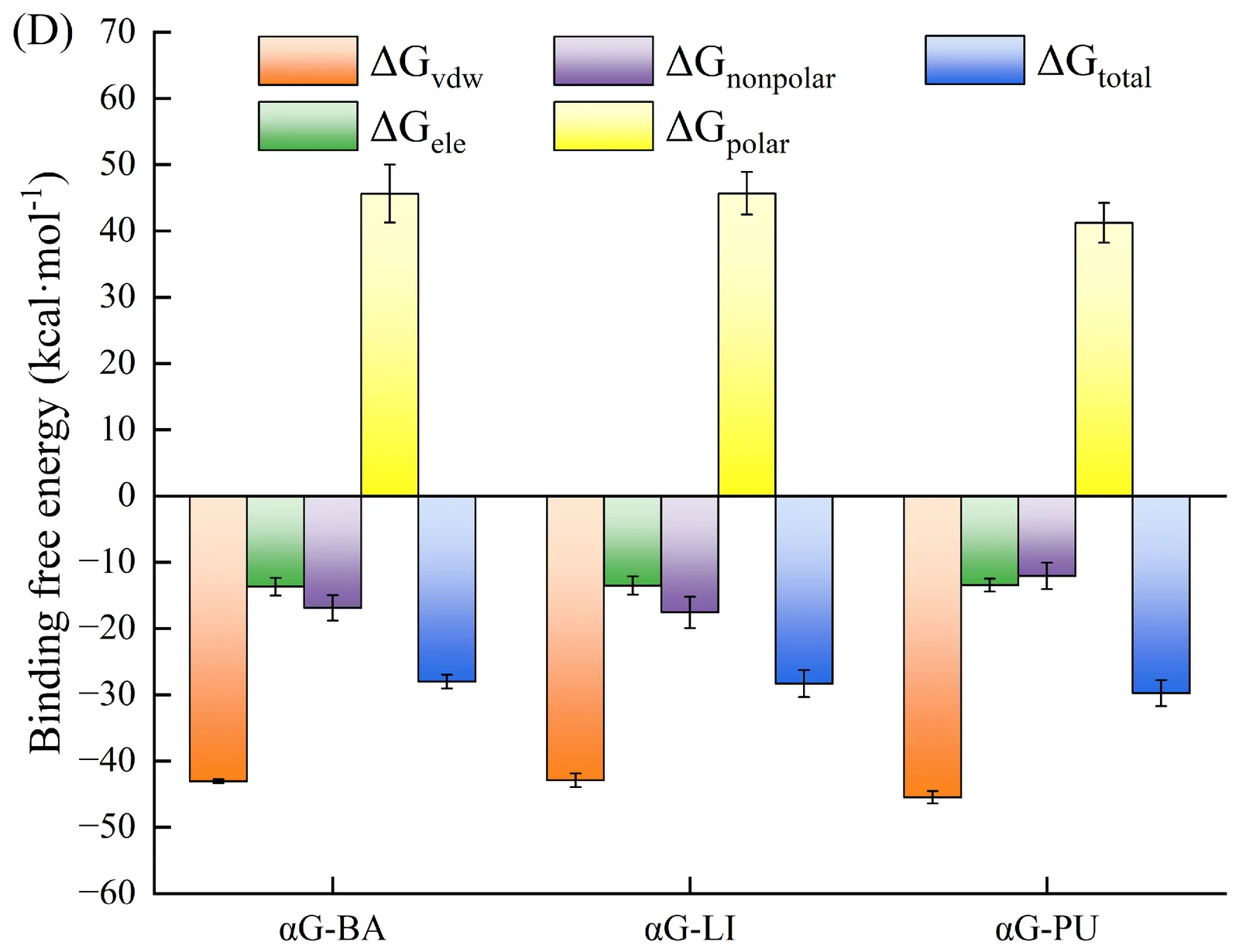
Number of hydrogen bonds (**A**,**B**) and binding free energy (**C**,**D**) resulting from the interactions between three flavonoids and αA/αG during molecular dynamics simulation.

Hydrogen bond formation plays an important role in maintaining the conformational stability of protein-ligand complexes [48]. As shown in Figure 8, αA-flavonoids (A) and αG-flavonoids (B) formed continuous hydrogen bonds during MD simulation. Persistent hydrogen bonds throughout the simulation generally indicate stronger ligand-binding capability. The number and stability of hydrogen bonds reflected the interaction strength and affinity between ligands and proteins. Specifically, during the 100 ns MD simulation, enzyme-BA complexes formed 1–3 hydrogen bonds, enzyme-LI complexes formed 1–2 hydrogen bonds, and enzyme-PU complexes formed 1–4 hydrogen bonds. Compared with BA and LI, PU exhibited a higher hydrogen-bonding capacity with both enzymes. This might be attributed to the abundant hydroxyl groups and glycosidic structure of PU, which facilitated the formation of more stable and diverse hydrogen-bond networks with enzyme residues, thereby enhancing the binding stability between PU and protein.

Binding free energy (ΔG) was calculated to determine the dominant driving forces involved in protein-ligand interactions [49]. As shown in Figure 8, further decomposition of energy components revealed that van der Waals interactions (ΔG_vdW_) provided the largest favorable contribution among all energy terms in all complexes. The van der Waals contributions of αA-PU and αG-PU complexes were −46.93 and −45.46 kcal/mol, respectively, which were higher than those of other interaction components, indicating that van der Waals forces were the predominant driving force responsible for stabilizing the flavonoid-enzyme. Additionally, electrostatic interactions (ΔG_ele_) and nonpolar solvation energy (ΔG_nonpolar_) also contributed favorably to complex formation [50], and polar solvation energy (ΔG_polar_) showed an unfavorable contribution to the binding process [51]. Nevertheless, the unfavorable ΔG_polar_ effect was compensated by the strong contributions from van der Waals interactions and hydrophobic interactions, thereby maintaining the overall spontaneity and stability of flavonoid-enzyme binding [41].

### 3.10. Investigation of Inhibition Mechanisms of BA/LI/PU Against αA and αG

**Figure 9:**
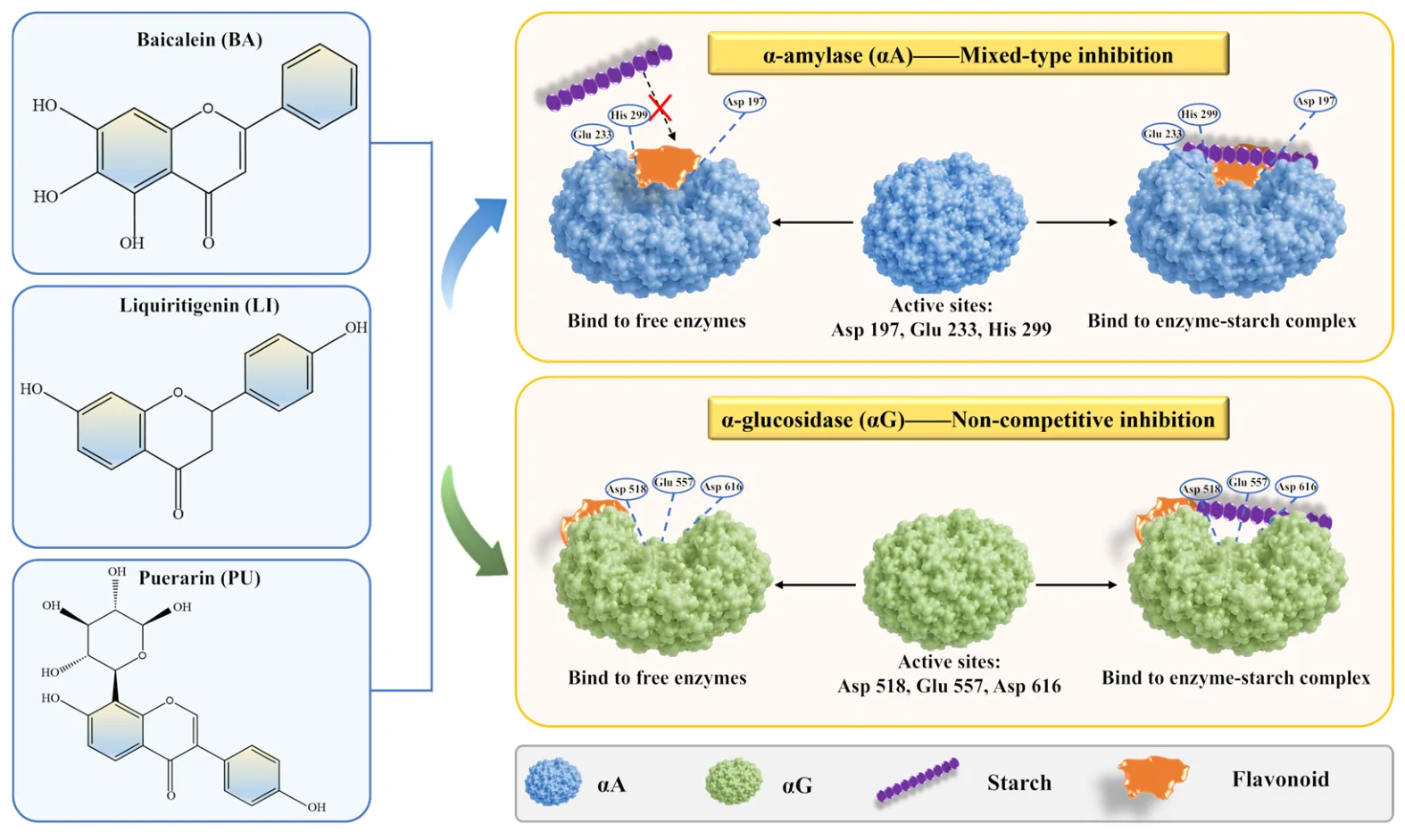
Schematic illustration of the molecular mechanisms underlying the inhibitory effects of BA/LI/PU against αA/αG.

The inhibitory mechanism of BA/LI/PU against αA and αG was exhibited in Figure 9. During the interaction between flavonoids and digestive enzymes, BA, LI, and PU primarily bound to αA and αG through non-covalent interactions, particularly hydrogen bonding and van der Waals forces, resulting in the spontaneous formation of stable enzyme-flavonoid complexes. As the concentration of flavonoids increased, catalytic activities of both enzymes were significantly inhibited, with PU exhibiting the strongest inhibitory effect, followed by BA and LI. For αA, three flavonoids exhibited a mixed-type competitive inhibition. The flavonoids could not only enter the active-site cavity of αA and interact with residues involved in substrate binding, thereby competing with starch for catalytic regions, but also bind to the αA-starch complex, leading to inhibition at multiple stages of the starch hydrolysis process [15,28]. This inhibitory mechanism aligned well with the findings of Xu et al. [19], who similarly confirmed that buckwheat hull polyphenols exerted mixed-type inhibition against αA while inducing protein unfolding and active pocket shielding. Similarly, Sun et al. [20] reported that bound polyphenols extracted from mung bean coat dietary fiber exerted a mixed-type inhibition against porcine pancreatic αA while driving protein structural changes and catalytic active site shielding. In contrast, the inhibition of αG by flavonoids was characterized as noncompetitive inhibition. The flavonoids preferentially interacted with regions outside the catalytic center of αG, rather than directly occupying the catalytic pocket [17,43]. Such binding behavior did not primarily interfere with substrate accessibility but instead induced conformational rearrangements of the enzyme structure, consequently reducing its catalytic efficiency. The binding of flavonoids also induced notable conformational alterations in both enzymes. Specifically, the microenvironment surrounding aromatic amino acid residues became less polar and more hydrophobic after flavonoid binding, suggesting that these residues were gradually buried within the hydrophobic core of the protein structure [15]. Moreover, the secondary structures of the enzymes underwent a transition from relatively flexible random coil regions toward more ordered α-helix and β-sheet structures, indicating an increase in structural compactness and conformational organization [35]. Molecular dynamics simulations further demonstrated that the flavonoid-enzyme complexes remained stable throughout the simulation period. The persistent hydrogen bonds formed during binding, together with the dominant contribution of van der Waals interactions to the binding energy, played crucial roles in maintaining complex stability [6]. Among the three flavonoids, PU formed the most extensive hydrogen-bonding network with both enzymes, resulting in stronger binding affinity and more pronounced conformational regulation. The consistency among binding strength, conformational modulation, and enzymatic inhibition further confirmed the structure-dependent inhibitory mechanism of flavonoids. Notably, the superior inhibitory activity of PU could be attributed to its unique structural characteristics, particularly the presence of glycosidic moieties and abundant hydroxyl groups, which provide additional interaction sites for hydrogen bond formation and facilitate the establishment of stronger and more diverse interactions with enzyme residues. Consequently, PU exhibited the most stable binding behavior and the highest inhibitory efficiency against both digestive enzymes, followed by BA and LI.

## 4. Conclusions

This study concentrated on investigating the inhibitory effects of three structurally distinct flavonoids (BA, LI and PU) against αA/αG and their underlying molecular mechanisms. The results showed that all three flavonoids exhibited dose-dependent inhibitory activity against αA and αG, with the order of PU > BA > LI, and PU displayed the strongest inhibitory capacity toward both enzymes. Kinetic analyses demonstrated that the three flavonoids inhibited αA in a mixed-type manner by embedding into the active-site cavity, whereas they non-competitively inhibited αG by binding to the peripheral allosteric region outside the catalytic pocket. Fluorescence, UV-Vis, CD, and FTIR analyses confirmed that the flavonoids statically quenched the intrinsic fluorescence of the enzymes, enhanced the hydrophobicity of the microenvironment surrounding aromatic amino acid residues, and promoted the transition of secondary structure from random coil to α-helix and β-sheet, thereby disrupting the native conformation required for efficient catalysis. Thermodynamic parameters and molecular dynamics simulations further verified that the binding was spontaneous and stabilized predominantly by hydrogen bonding and van der Waals forces, with PU forming the most abundant hydrogen-bond network and exhibiting the highest conformational stability. Collectively, these results reveal a structure-dependent, enzyme-specific non-covalent inhibition mechanism of flavonoids against starch-digesting enzymes. Notably, abundant hydroxyl groups of PU provided additional interaction sites for hydrogen-bond formation, contributing to its superior inhibitory efficiency. Overall, these findings established a relationship between interaction and inhibition for flavonoids toward αA/αG and provided molecular insights into the structural basis governing their enzyme-specific inhibitory behaviors.

## Figures and Tables

**Table 1:** IC_50_ values and inhibition kinetic parameters for the inhibition of α-amylase and α-glucosidase by BA, LI, and PU.

Enzymes	Flavonoids	[I] (mg/mL)	V_m_ (mg/mL·min)	K_m_ (mg/mL)	IC_50_ (mg/mL)	K_ic_ (mg/mL)	K_iu_ (mg/mL)	Inhibition Type
αA	BA	0	0.066 ± 0.001 ^ab^	7.843 ± 0.192 ^b^	1.178 ± 0.028 ^b^	0.635 ± 0.087 ^b^	0.712 ± 0.052 ^b^	Mix
		0.3	0.058 ± 0.002 ^bc^	7.297 ± 0.183 ^b^				
		0.6	0.057 ± 0.001 ^bc^	6.554 ± 0.093 ^bc^				
		0.9	0.041 ± 0.001 ^d^	5.894 ± 0.219 ^c^				
	LI	0	0.092 ± 0.004 ^a^	10.472 ± 0.083 ^a^	1.411 ± 0.033 ^a^	0.672 ± 0.098 ^a^	0.791 ± 0.088 ^a^	Mix
		0.3	0.088 ± 0.002 ^a^	8.657 ± 0.283 ^b^				
		0.6	0.071 ± 0.001 ^ab^	6.943 ± 0.193 ^bc^				
		0.9	0.064 ± 0.005 ^b^	4.377 ± 0.173 ^d^				
	PU	0	0.052 ± 0.001 ^c^	5.559 ± 0.261 ^c^	0.909 ± 0.017 ^c^	0.594 ± 0.053 ^c^	0.677 ± 0.032 ^c^	Mix
		0.3	0.036 ± 0.001 ^d^	5.679 ± 0.193 ^c^				
		0.6	0.028 ± 0.003 ^e^	4.869 ± 0.103 ^d^				
		0.9	0.017 ± 0.002 ^f^	4.841 ± 0.118 ^d^				
αG	BA	0	0.075 ± 0.012 ^ab^	11.066 ± 0.093 ^b^	2.691 ± 0.187 ^b^	—	0.352 ± 0.011 ^a^	NC
		0.3	0.062 ± 0.011 ^b^	11.362 ± 1.032 ^b^				
		0.6	0.061 ± 0.009 ^b^	11.651 ± 1.293 ^b^				
		0.9	0.055 ± 0.004 ^b^	11.210 ± 0.023 ^ab^				
	LI	0	0.088 ± 0.002 ^a^	12.852 ± 0.093 ^a^	3.588 ± 0.293 ^a^	—	0.356 ± 0.021 ^a^	NC
		0.3	0.082 ± 0.019 ^a^	12.827 ± 0.076 ^a^				
		0.6	0.077 ± 0.002 ^ab^	12.547 ± 0.028 ^a^				
		0.9	0.073 ± 0.007 ^ab^	12.669 ± 1.392 ^a^				
	PU	0	0.052 ± 0.004 ^c^	11.091 ± 1.382 ^b^	1.524 ± 0.133 ^c^	—	0.326 ± 0.023 ^b^	NC
		0.3	0.051 ± 0.006 ^c^	11.108 ± 0.038 ^b^				
		0.6	0.043 ± 0.001 ^d^	11.247 ± 0.084 ^b^				
		0.9	0.042 ± 0.003 ^d^	11.247 ± 0.091 ^b^				

Note: Data were means ± SD (n ≥ 3), values in the same column of the same category with different letters differ significantly (*p* < 0.05).

**Table 2:** Quenching parameters of interactions between αA/αG with BA/LI/PU at different temperatures.

Enzymes	Flavonoids	T (K)	K_sv_ (×10^3^ M^−1^)	K_q_ (×10^11^ M^−1^·s^−1^)	K_a_	n	△H (KJ·mol^−1^)	△S (J·mol^−1^·K^−1^)	△G (KJ·mol^−1^)
αA	BA	298.15	9.350	9.350	1.031 × 10^6^	1.07	−78.10	−49.36	−63.38
		303.15	7.918	7.918	1.744 × 10^6^	1.11			−63.14
		310.15	6.864	6.864	5.473 × 10^6^	1.10			−62.79
	LI	298.15	5.996	5.996	4.674 × 10^5^	1.92	−32.39	−27.74	−24.12
		303.15	5.330	5.330	1.725 × 10^5^	0.93			−23.98
		310.15	3.818	3.818	6.916 × 10^5^	1.04			−23.79
	PU	298.15	20.820	20.820	2.375 × 10^6^	1.09	−52.01	−52.43	−36.38
		303.15	15.199	15.199	3.094 × 10^6^	1.11			−36.12
		310.15	13.408	13.408	5.307 × 10^6^	1.06			−35.75
αG	BA	298.15	5.432	5.432	8.731 × 10^3^	0.83	−64.22	−47.38	−50.09
		303.15	2.567	2.567	2.128 × 10^4^	0.83			−49.86
		310.15	1.703	1.703	2.484 × 10^4^	0.84			−49.53
	LI	298.15	4.331	4.331	3.218 × 10^3^	0.81	−51.20	−37.38	−40.06
		303.15	2.229	2.229	4.968 × 10^3^	0.77			−39.87
		310.15	0.692	0.692	7.218 × 10^3^	0.80			−39.61
	PU	298.15	9.660	9.660	1.397 × 10^5^	0.95	−37.22	−23.48	−30.22
		303.15	6.954	6.954	1.919 × 10^5^	0.95			−30.10
		310.15	6.787	6.787	2.514 × 10^5^	0.97			−29.94

**Table 3:** The results of the binding energy (E_b_) of BA, LI, and PU to αA and αG.

Enzymes	Flavonoids	E_b_ (kcal/mol)
αA	BA	−7.79
	LI	−8.11
	PU	−9.11
αG	BA	−8.89
	LI	−8.74
	PU	−9.87

## Data Availability

The original contributions presented in this study are included in the article/Supplementary Materials. Further inquiries can be directed to the corresponding author.

## Supplementary Materials

The following supporting information can be downloaded at: https://www.mdpi.com/article/10.3390/foods15203731/s1, Figure S1: Substrate concentration-time curve used to determine initial reaction rates of αA/αG in the presence of BA/LI/PU; Figure S2: Nonlinear fitting of competitive, noncompetitive, and mixed inhibition models for BA/LI/PU against αA/αG; Table S1: Statistical comparison of competitive, pure noncompetitive, and mixed inhibition models for BA/LI/PU against αA/αG.
