# Supplementary material for: Unraveling Inhibition Mechanism of Structurally Distinct Flavonoids Against α-Amylase and α-Glucosidase: Insights from Multi-Spectroscopic and In Silico Analyses
Source: Foods. 2026 Oct 20;15(20):3731. doi: 10.3390/foods15203731 (PMC13704501; doi:10.3390/foods15203731)
Supplement: Supplementary file 1 [file foods-15-03731-s001.zip › foods-4570538-supplementary.pdf]

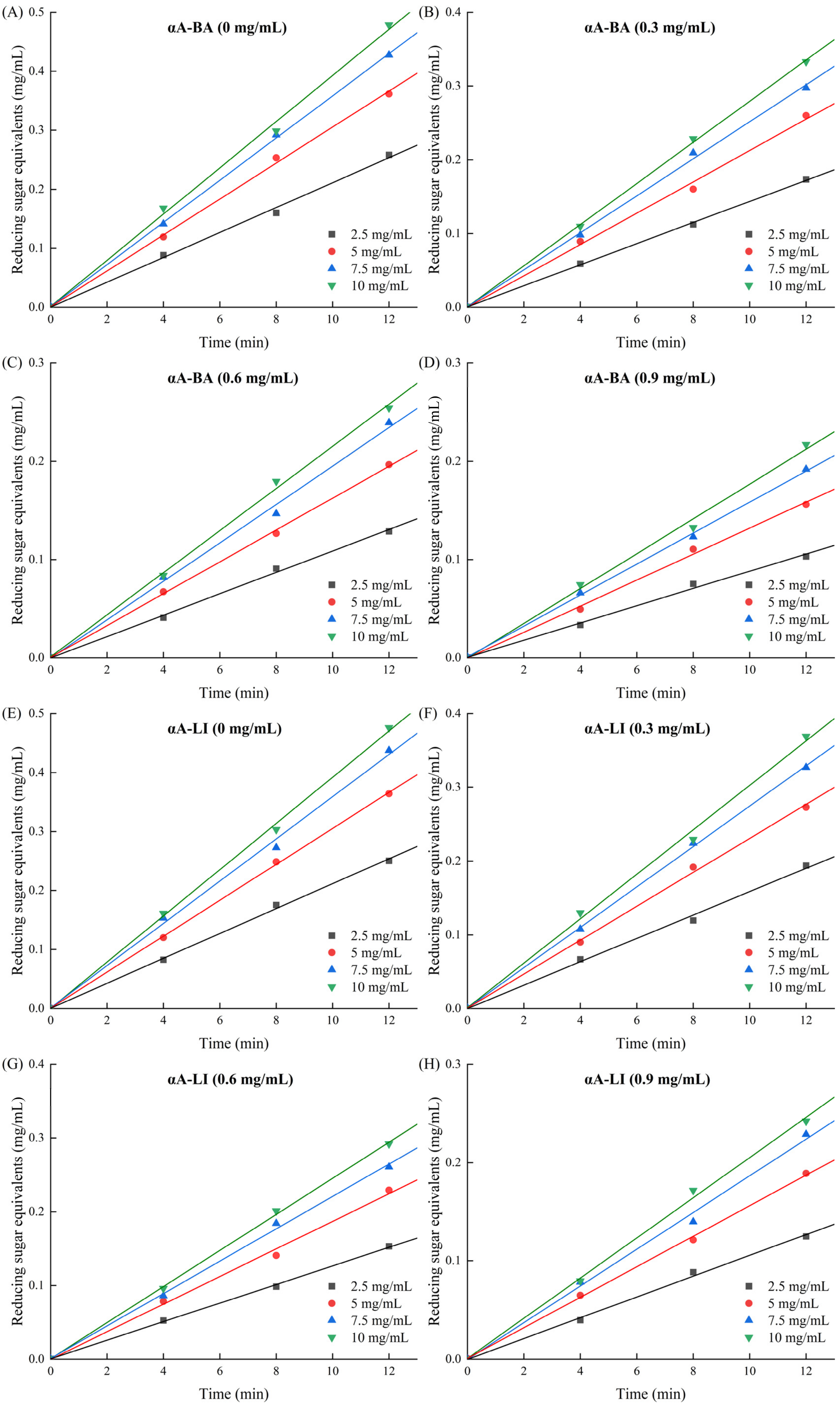

(A)
αA-BA (0 mg/mL)
(B)
αA-BA (0.3 mg/mL)
(C)
αA-BA (0.6 mg/mL)
(D)
αA-BA (0.9 mg/mL)
(E)
αA-LI (0 mg/mL)
(F)
αA-LI (0.3 mg/mL)
(G)
αA-LI (0.6 mg/mL)
(H)
αA-LI (0.9 mg/mL)
Reducing sugar equivalents (mg/mL)
Time (min)
2.5 mg/mL
5 mg/mL
7.5 mg/mL
10 mg/mL

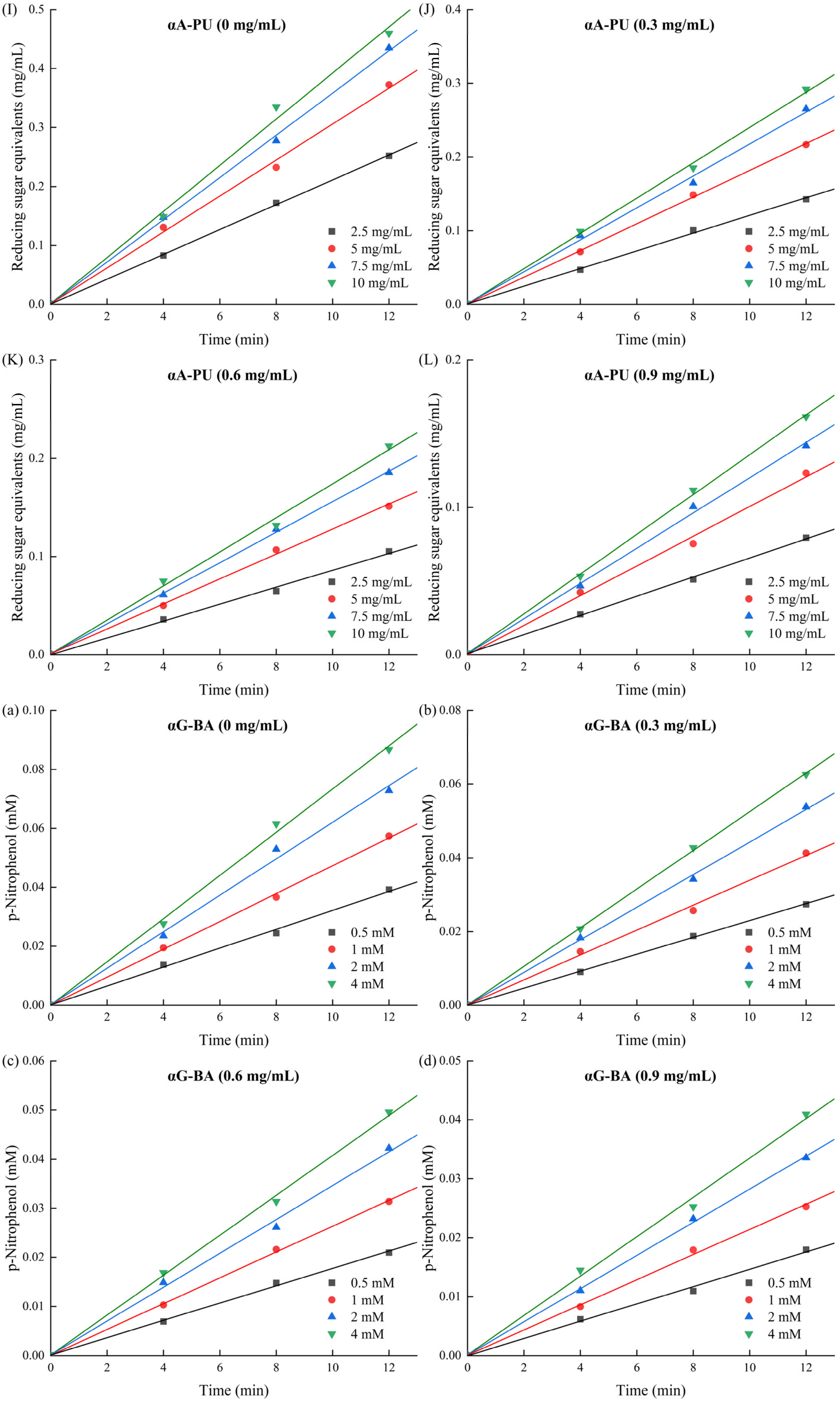

(I)
αA-PU (0 mg/mL)
Reducing sugar equivalents (mg/mL)
Time (min)
2.5 mg/mL
5 mg/mL
7.5 mg/mL
10 mg/mL
(J)
αA-PU (0.3 mg/mL)
Reducing sugar equivalents (mg/mL)
Time (min)
2.5 mg/mL
5 mg/mL
7.5 mg/mL
10 mg/mL
(K)
αA-PU (0.6 mg/mL)
Reducing sugar equivalents (mg/mL)
Time (min)
2.5 mg/mL
5 mg/mL
7.5 mg/mL
10 mg/mL
(L)
αA-PU (0.9 mg/mL)
Reducing sugar equivalents (mg/mL)
Time (min)
2.5 mg/mL
5 mg/mL
7.5 mg/mL
10 mg/mL
(a)
αG-BA (0 mg/mL)
p-Nitrophenol (mM)
Time (min)
0.5 mM
1 mM
2 mM
4 mM
(b)
αG-BA (0.3 mg/mL)
p-Nitrophenol (mM)
Time (min)
0.5 mM
1 mM
2 mM
4 mM
(c)
αG-BA (0.6 mg/mL)
p-Nitrophenol (mM)
Time (min)
0.5 mM
1 mM
2 mM
4 mM
(d)
αG-BA (0.9 mg/mL)
p-Nitrophenol (mM)
Time (min)
0.5 mM
1 mM
2 mM
4 mM

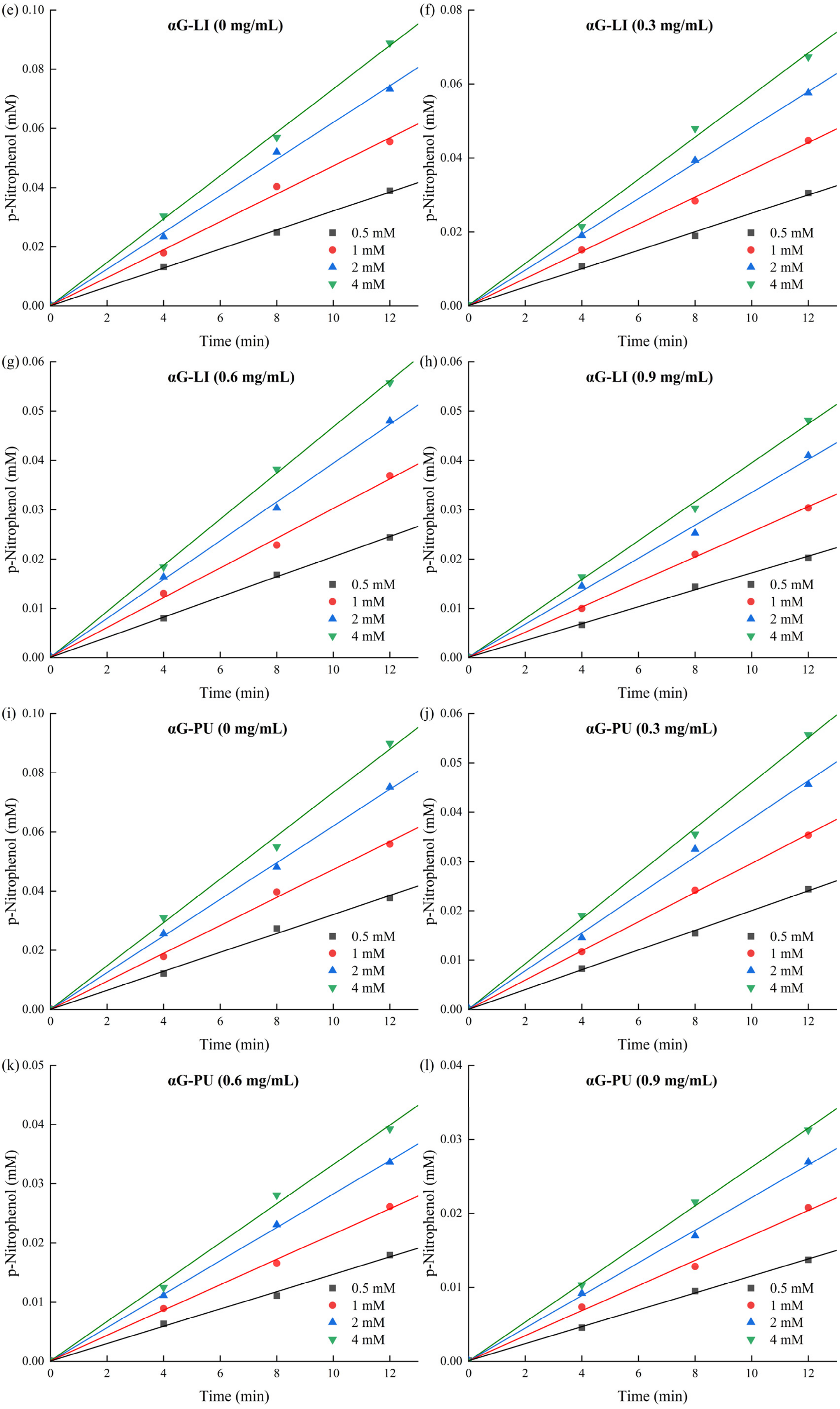
(e)
αG-LI (0 mg/mL)
(f)
αG-LI (0.3 mg/mL)
(g)
αG-LI (0.6 mg/mL)
(h)
αG-LI (0.9 mg/mL)
(i)
αG-PU (0 mg/mL)
(j)
αG-PU (0.3 mg/mL)
(k)
αG-PU (0.6 mg/mL)
(l)
αG-PU (0.9 mg/mL)
p-Nitrophenol (mM)
Time (min)
0.5 mM
1 mM
2 mM
4 mM

Figure S1. Substrate concentration-time curve used to determine initial reaction rates of αA/αG in the presence of BA/LI/PU.

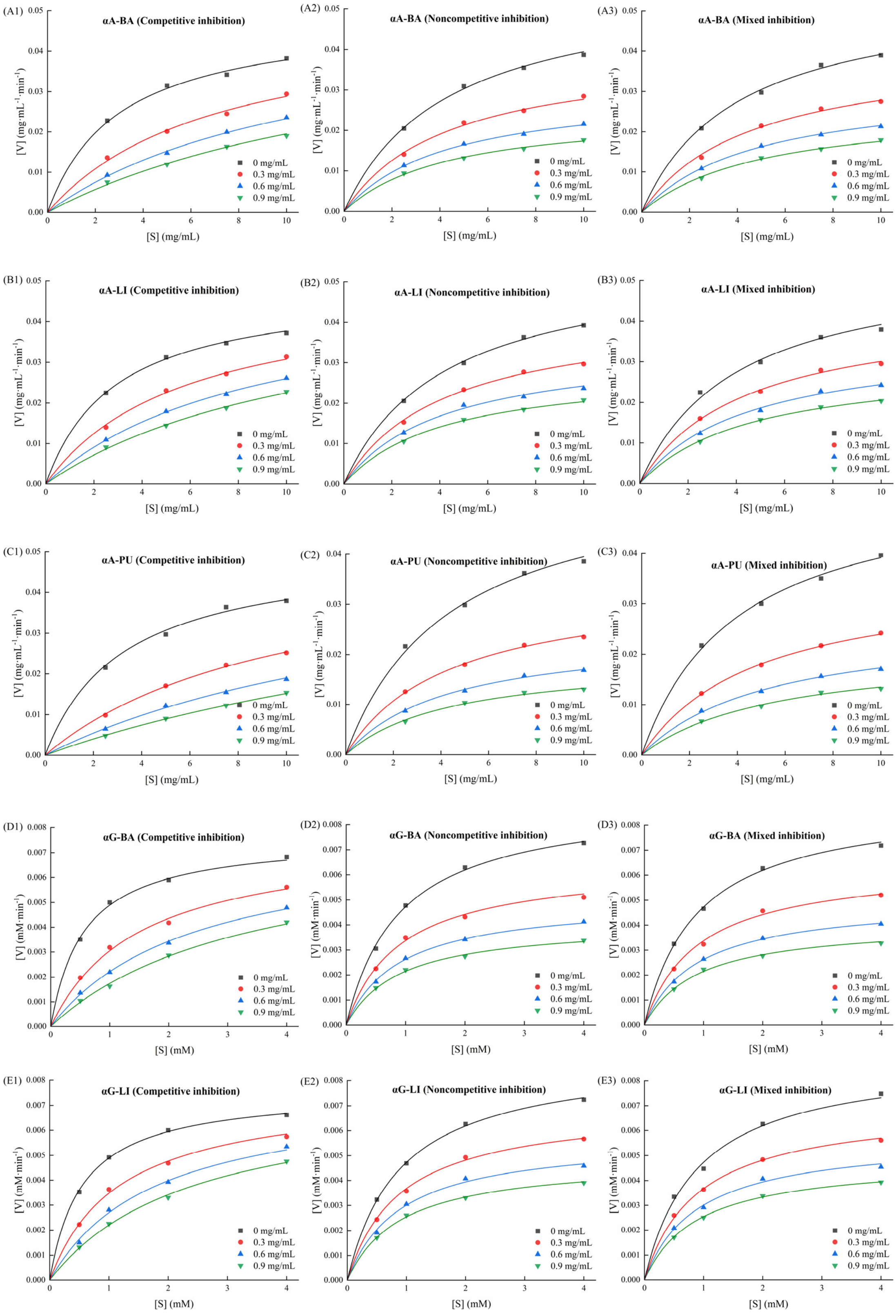
(A1) αA-BA (Competitive inhibition)
(A2) αA-BA (Noncompetitive inhibition)
(A3) αA-BA (Mixed inhibition)
(B1) αA-LI (Competitive inhibition)
(B2) αA-LI (Noncompetitive inhibition)
(B3) αA-LI (Mixed inhibition)
(C1) αA-PU (Competitive inhibition)
(C2) αA-PU (Noncompetitive inhibition)
(C3) αA-PU (Mixed inhibition)
(D1) αG-BA (Competitive inhibition)
(D2) αG-BA (Noncompetitive inhibition)
(D3) αG-BA (Mixed inhibition)
(E1) αG-LI (Competitive inhibition)
(E2) αG-LI (Noncompetitive inhibition)
(E3) αG-LI (Mixed inhibition)
[V] (mg·mL⁻¹·min⁻¹)
[S] (mg/mL)
[V] (mM·min⁻¹)
[S] (mM)
0 mg/mL
0.3 mg/mL
0.6 mg/mL
0.9 mg/mL

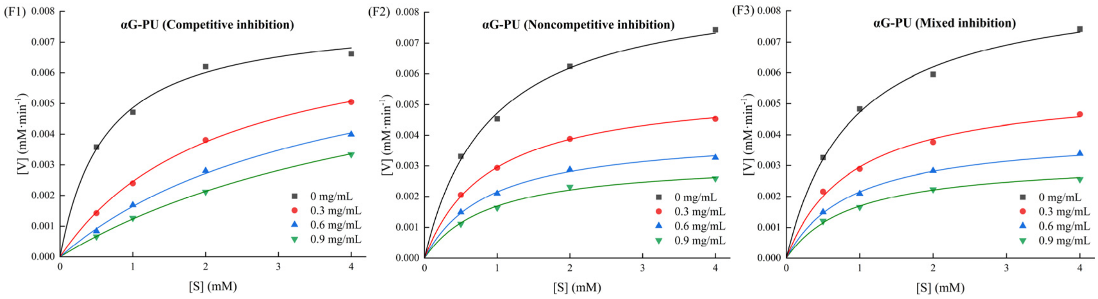

Figure S2. Nonlinear fitting of competitive, noncompetitive, and mixed inhibition models for BA/LI/PU against αA/αG.

Table S1. Statistical comparison of competitive, pure noncompetitive, and mixed inhibition models for BA/LI/PU against αA/αG.

System	Model	R^2	SSE	AICc	ΔAICc
αA-BA	Competitive	0.979048	2.348×10^{-5}	-206.91	88.47
αA-BA	Noncompetitive	0.999300	7.840×10^{-7}	-261.30	34.07
αA-BA	Mixed	0.999934	7.427×10^{-8}	-295.37	0.00
αA-LI	Competitive	0.976730	2.256×10^{-5}	-207.55	84.59
αA-LI	Noncompetitive	0.999608	3.802×10^{-7}	-272.88	19.26
αA-LI	Mixed	0.999906	9.091×10^{-8}	-292.14	0.00
αA-PU	Competitive	0.982896	2.360×10^{-5}	-206.83	85.93
αA-PU	Noncompetitive	0.999178	1.134×10^{-6}	-255.40	37.36
αA-PU	Mixed	0.999937	8.746×10^{-8}	-292.76	0.00
αG-BA	Competitive	0.926982	2.850×10^{-6}	-240.65	128.51
αG-BA	Noncompetitive	0.999976	9.258×10^{-10}	-369.17	0.00
αG-BA	Mixed	0.999978	8.772×10^{-10}	-366.39	2.77
αG-LI	Competitive	0.936950	2.265×10^{-6}	-244.33	125.76
αG-LI	Noncompetitive	0.999976	8.737×10^{-10}	-370.09	0.00
αG-LI	Mixed	0.999976	8.633×10^{-10}	-366.65	3.44
αG-PU	Competitive	0.925860	3.273×10^{-6}	-238.44	132.17
αG-PU	Noncompetitive	0.999981	8.460×10^{-10}	-370.61	0.00
αG-PU	Mixed	0.999981	8.402×10^{-10}	-367.08	3.53
